# p53 and TAp63 Promote Keratinocyte Proliferation and Differentiation in Breeding Tubercles of the Zebrafish

**DOI:** 10.1371/journal.pgen.1004048

**Published:** 2014-01-09

**Authors:** Boris Fischer, Manuel Metzger, Rebecca Richardson, Philipp Knyphausen, Thomas Ramezani, Rainer Franzen, Elmon Schmelzer, Wilhelm Bloch, Thomas J. Carney, Matthias Hammerschmidt

**Affiliations:** 1 Institute of Developmental Biology, University of Cologne, Cologne, Germany; 2 Cell Biology, Max Planck Institute for Plant Breeding Research, Cologne, Germany; 3 Institute of Cardiology and Sports Medicine, German Sport University Cologne, Cologne, Germany; 4 Institute of Molecular and Cell Biology, Proteos, Singapore; 5 Center for Molecular Medicine Cologne, University of Cologne, Cologne, Germany; 6 Cologne Excellence Cluster on Cellular Stress Responses in Aging-Associated Diseases, University of Cologne, Cologne, Germany; University of Colorado Health Sciences Center, United States of America

## Abstract

p63 is a multi-isoform member of the p53 family of transcription factors. There is compelling genetic evidence that ΔNp63 isoforms are needed for keratinocyte proliferation and stemness in the developing vertebrate epidermis. However, the role of TAp63 isoforms is not fully understood, and TAp63 knockout mice display normal epidermal development. Here, we show that zebrafish mutants specifically lacking TAp63 isoforms, or p53, display compromised development of breeding tubercles, epidermal appendages which according to our analyses display more advanced stratification and keratinization than regular epidermis, including continuous desquamation and renewal of superficial cells by derivatives of basal keratinocytes. Defects are further enhanced in TAp63/p53 double mutants, pointing to partially redundant roles of the two related factors. Molecular analyses, treatments with chemical inhibitors and epistasis studies further reveal the existence of a linear TAp63/p53->Notch->caspase 3 pathway required both for enhanced proliferation of keratinocytes at the base of the tubercles and their subsequent differentiation in upper layers. Together, these studies identify the zebrafish breeding tubercles as specific epidermal structures sharing crucial features with the cornified mammalian epidermis. In addition, they unravel essential roles of TAp63 and p53 to promote both keratinocyte proliferation and their terminal differentiation by promoting Notch signalling and caspase 3 activity, ensuring formation and proper homeostasis of this self-renewing stratified epithelium.

## Author Summary

The mammalian epidermis is a stratified self-renewing epithelium, in which cell loss at the surface is properly balanced by cell proliferation in basal layers to ensure tissue homeostasis. But how is this balance genetically controlled? Here, we address this question in zebrafish breeding tubercles, epidermal appendages in which keratinocytes undergo more advanced differentiation processes than in regular fish epidermis, sharing crucial features with the cornified mammalian skin. We identify a linear pathway consisting of the transcription factor p53 and its close relative TAp63, which activate Notch signalling and thereby caspase 3 to promote terminal differentiation and eventual shedding of keratinocytes in upper tubercle layers, while at the same time employing non-cell autonomous mechanisms to promote keratinocyte proliferation at the tubercle base, thereby ensuring proper development and homeostasis of this self-renewing tissue. Such a two-fold function of the pathway is consistent with the formerly reported dual role of a caspase during wing regeneration in the fruitfly. Our findings will help to better understand the seemingly contrary effects described for TAp63 in different mammalian systems, while demonstrating partial functional redundancy between p53 and TAp63 during epidermal development in fish.

## Introduction

The mammalian epidermis is a self-renewing stratified epithelium on the outer surface of the skin. During embryogenesis, it develops from the surface ectoderm, which is initially a single-layered epithelium. Stratification is initiated (E10 in mouse) with the formation of the outer periderm, leading to a bi-layered epidermal organization in which peridermal cells are attached to each other via tight junctions to protect the embryo against the amniotic fluid [1], [2]. Further epidermal maturation (E12.5–E17.5 in mouse) takes place beneath with the consecutive formation of the spinous, granular and cornified layers, establishing the later epidermal barrier, while the periderm is sloughed off [3]–[6]. Crucial contributions to this later epidermal barrier come from the granular layer, in which cells are sealed to each other via tight junctions, and from the outer cornified layers, providing physical resistance and preventing dehydration. Formation of this water barrier is essential for the adaptation to terrestrial life both during the ontogeny [7] and the evolution [8], [9] of land-based vertebrates. Accordingly, fish lack epidermal cornification.

In zebrafish, the embryonic and larval epidermis is bi-layered, consisting of an outer enveloping layer (EVL) segregating from inner cells before surface ectoderm specification [10], [11], and a basal keratinocyte layer, resembling the bi-layered organization of the mammalian epidermis at midgestation stages. Further stratification of the zebrafish epidermis only commences with the onset of metamorphosis (after three weeks of development), during which the epidermis becomes multi-layered. However, keratinocytes remain metabolically active throughout, including the outer-most layer, and lack morphological signs of cornification [12], [13]. This is in contrast to the organization of breeding tubercles, contact and secondary sex organs on the head and pectoral fin rays. Based on morphological studies in several other fish species, they were described as “keratinized epidermal appendages covered by a layer of dead cells with altered content” [14], suggesting that in breeding tubercles, keratinocytes might undergo more advanced, cornification-like differentiation processes.

Mammalian keratinocyte cornification is a multi-step process initiated by a switch in the expression of particular keratin genes, followed by the expression of the keratin-bundling protein filaggrin and proteins such as involucrin and loricrin, which together with keratins become cross-linked by transglutaminases (Tgm1-4) to reinforce the formation of a cornified envelope [15]–[17]. Furthermore, lipids stored in lamellar bodies are extruded into the extracellular space to form a lipid envelope. During these later steps, cells enter an apoptotic-like phase, lose cytoplasmic organelles including the nucleus, and are eventually sloughed from the skin surface. This loss of corneocytes by desquamation is tightly balanced by keratinocyte proliferation in basal layers, allowing constant self-renewal during epidermal homeostasis.

A key regulator of mammalian epidermal stratification and keratinocyte proliferation and differentiation is p63, a homolog of the tumour suppressor and transcription factor p53 [18]–[24]. Use of alternative promoters of the *Tp63* gene gives rise to two isoform categories: those with an N-terminal transactivation domain (TAp63 isoforms), as also present in p53, and those lacking this domain (ΔNp63 isoforms) and acting as inhibitory competitors of their TA counterparts. In addition, alternative splicing gives rise to at least three different C-terminal isoforms (α,β,γ) in each category, and to different N-terminal isoforms of TAp63 [25]. Recent analyses of mice specifically lacking the ΔN isoforms indicate that ΔNp63 is required for maintaining the proliferative potential of basal keratinocytes in embryonic epidermis while preventing their premature entry into terminal differentiation [26], consistent with findings obtained in cultured keratinocytes [27]–[29]. In contrast, TAp63 and p53 might promote keratinocyte differentiation. Thus, in inducible cell lines, TAp63 activates genes involved in keratinocyte differentiation, including different *tgm*s [30]. Also p53, via direct and indirect activation of Notch signalling, has been shown to be required for keratinocyte differentiation in cell culture systems and during squamous cell carcinoma suppression in mouse tumour models [31], [32]. siRNA-mediated knockdown studies in organotypic cultures of human keratinocytes further suggest that it is primarily p53 that antagonizes the proliferation-stimulating effect of ΔNp63, and that the contribution of TAp63 to keratinocyte differentiation is minor compared to that of ΔNp63 [29], [33]. Furthermore, TA-specific p63 mutant mice lack an abrogation of keratinocyte differentiation [34]–[36], leaving the *in vivo* role of TAp63 during keratinocyte development unclear.

Notch signalling promotes different steps of mammalian keratinocyte differentiation *in vivo* [37]–[41]. Upon binding of Delta or Jagged ligands, the Notch receptor is cleaved and releases its intracellular domain (NICD), which binds to the transcriptional repressor RBP-J, enabling it to activate target genes that are repressed in the absence of Notch signals. Findings concerning the interconnection between p53/p63 and Notch signalling are controversial. Thus, *Jagged1/2* and *Notch1* have been reported to be positively regulated by p53 and TAp63γ [31], [42]–[46], but also by ΔNp63 [26], [43], while in other systems, the Notch target *Hes1* was negatively regulated by both TAp63 and ΔNp63 [43]. How Notch signalling promotes keratinocyte differentiation is also incompletely understood [38], [47]. One described Notch/RBP-target gene in embryonic mouse keratinocytes encodes caspase 3 [39]. This cysteine protease not only executes apoptosis, but also promotes terminal differentiation processes in a range of cell types [48], leading to delayed basal keratinocyte differentiation in *caspase 3* mutant mouse embryos [39].

Here, we identify the zebrafish breeding tubercles as sites with higher keratinocyte proliferation in basal layers as well as more advanced, cornification-like keratinocyte differentiation in upper layers, including transglutaminase expression and stronger keratinization, exclusive presence of tight junctions in second-tier keratinocytes, and rudimentary lipid envelope formation and constant desquamation and renewal of surface keratinocytes. In addition, we identify zebrafish TAp63 isoforms and a TA-specific p63 loss-of-function mutant, revealing essential and partially redundant roles of TAp63 and p53 to promote both keratinocyte proliferation at the base and terminal keratinocyte differentiation in upper layers of breeding tubercles. Both effects are mediated via Notch signalling and activated caspase 3, although these mediators are restricted to upper breeding tubercle layers, pointing to a combination of cell autonomous and non-cell autonomous effects. These findings will help to better understand the seemingly controversial roles described for TAp63 and p53 in different systems.

## Results

### Regular epidermis and breeding tubercles display different patterns of superficial cell renewal

**Figure 1:**
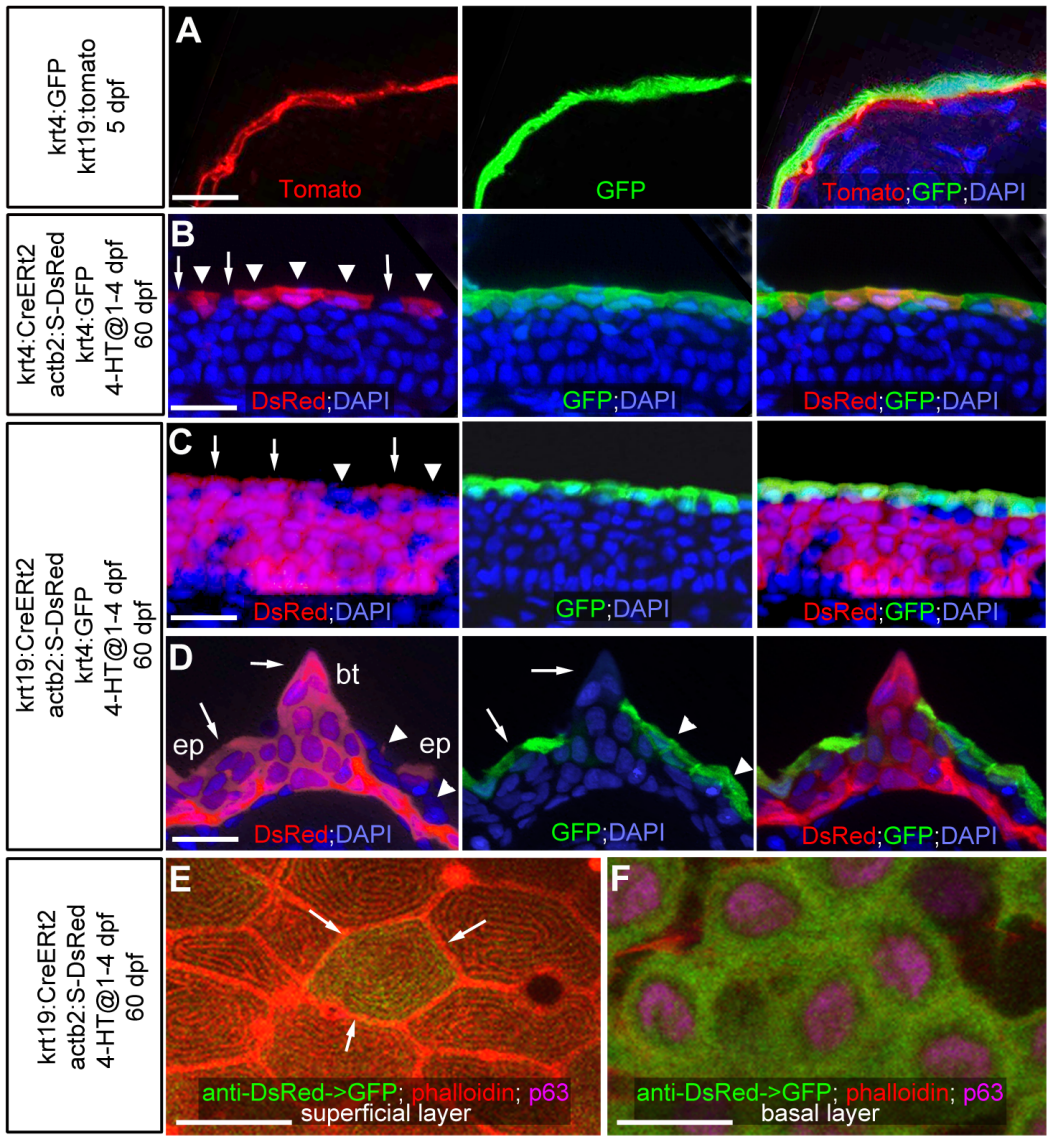
Transgenic lineage tracing of enveloping layer cells and basal keratinocytes in regular epidermis and breeding tubercles. (A–D) show transgene-encoded GFP and Tomato/dsRed fluorescence; transverse cryosections stained with DAPI; red channel on the left, green channel in the middle, merged channels on the right; (A) larva at 5 dpf; (B–D) juvenile fish at 60 dpf; (B,C) regular epidermis; (D) breeding tubercle on lower jaw. In (B–D), superficial cells deriving from the embryonic enveloping layer (EVL) are indicated with arrowheads, superficial cells deriving from the embryonic basal layer with arrows. (A) At 5 dpf, krt4-driven transgene expression is confined to EVL (in green), krt19-driven transgene expression to basal layer (in red). (B,C) At 60 dpf, derivatives of EVL cells (red; B) and basal keratinocytes (red, C) are present in the outer layer of regular epidermis, intermixed with unlabelled cells, but expressing the same marker gene (tg(krt4:GFP) in green). (D) At 60 dpf, the breeding tubercle (bt), including its superficial layer, consists solely of derivatives of basal cells (in red). However, superficial cells lack expression of krt4:GFP (green). (E,F) anti-DsRed (in green) and anti-p63 (in pink) immunofluorescences, combined with phalloidin-staining of cytoskeletal actin (in red); confocal images of superficial (E) and basal layer (F) of regular epidermis; 60 dpf. A clone of labelled basal cells (F; green) has given rise to a single superficial cell (marked by arrows in E), which displays the same microridge pattern as non-labelled neighbours. Scale bars: 20 µm (A–D), 10 µm (E). Abbreviations: 4-HT, 4-hydroxytamoxifen; act2b:S-DsRed, actb2:loxP-STOP-loxP-dsREDEx; bt, breeding tubercle; ep, regular epidermis.

Even after metamorphosis (approximately 30 days post fertilization; dpf), when the zebrafish epidermis has become multi-layered, superficial epidermal cells display crucial similarities to the embryonic enveloping layer (EVL), like the presence of tight junctions and distinct microridges at their outer surface [12]. To determine the developmental origin of superficial cells of the adult zebrafish epidermis, we carried out transgenic lineage tracing experiments, using promoter elements that drive embryonic expression confined to the outer EVL (*krt4*) [49] or the basal layer (*krt19*) [50], respectively (Figure 1A; 5 dpf). Employing these promoters in an inducible binary transgenic Cre/Lox-system, embryonic EVL cells or basal keratinocytes were stably labeled via tamoxifen application from 1–4 dpf (Figure 1B–F). At 60 dpf, superficial cells of the regular body epidermis consisted of a mixture of derivates of embryonic EVL cells (Figure 1B) and basal keratinocytes (Figure 1C,D). However, both expressed the same marker genes (Figure 1B–D; and data not shown) and displayed identical morphological characteristics (Figure 1E). This suggests that in regular epidermis, embryonic EVL cells can persist beyond metamorphosis and are only slowly replaced by derivatives of basal keratinocytes. However, renewed superficial cells seem to have the same properties and functions as persisting EVL cells.

A different pattern was observed in spike-like epidermal structures that according to their location and fine structure (see below) were identified as breeding tubercles [14]. Here, superficial cells consisted solely of derivatives of basal keratinocytes and failed to express markers of the embryonic EVL and of the superficial cells of the regular epidermis (Figure 1D; n = 13/13), suggesting that they have been renewed completely and have acquired a different fate.

### Breeding tubercles of adult zebrafish display more advanced stratification and keratinocyte differentiation

**Figure 2:**
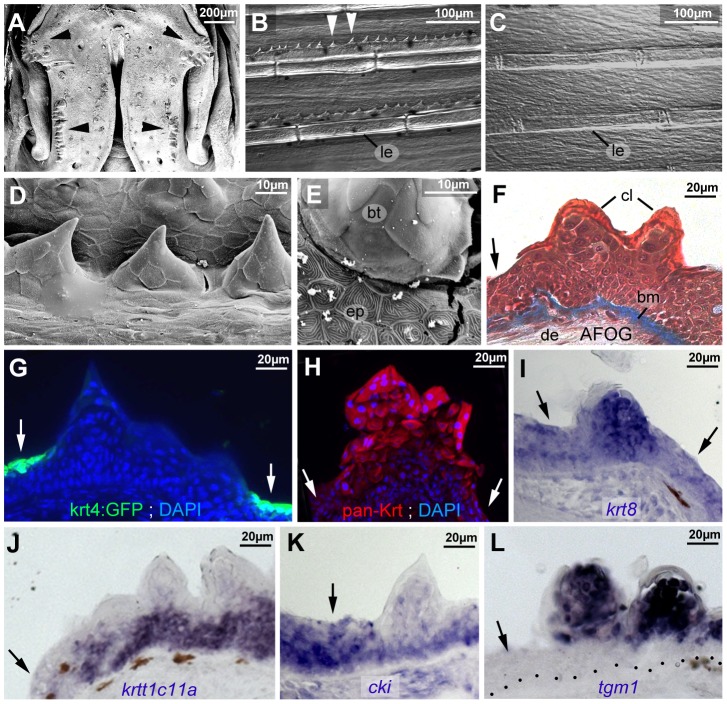
Breeding tubercle keratinocytes undergo more advanced keratinization. All images show breeding tubercles in wild-type zebrafish of one year of age; (A–E) views on body surface; (F–L) transverse sections through lower jaw. (A) Scanning electron micrograph (SEM) of the lower jaw; breeding tubercles are indicated by arrowheads. (B,C) Nomarski micrographs of pectoral fins of male (B) and female (C) fish. Bony rays (lepidotrichia) are marked (le). (D,E) SEM images of breeding tubercles (bt) on a male pectoral fin. (F) Trichrome (AFOG) staining; breeding tubercles consist of more cell layers than adjacent epidermis (arrow). The tubercle cap layer (cl) is stained in lighter red, resulting from its stronger keratinization. (G–L) GFP fluorescence of tg(krt4:GFP) line (G, green) and pan-type II Keratin immunofluorescence (H; red), in combination with DAPI staining. (I–L) in situ hybridization with indicated probes. Epidermis is indicated by arrows; dotted line in L marks basement membrane. Abbreviations: bm, basement membrane; bt, breeding tubercle; cl, cap layer; de, dermis; ep, regular epidermis; le, lepidotrichia.

In adult zebrafish, breeding tubercles were exclusively present in a disc- and row-like structure on each side of the lower jaw (Figure 2A), as well as in rows along the bony rays of the pectoral fins of males (Figure 2B), but not females (Figure 2C). Scanning electron microscopy (SEM) revealed that superficial cells of breeding tubercles, also called cap cells [14], lacked the microridges present at the outer surface of regular epidermis (Figure 2D,E). In addition, they lacked expression of *tg(krt4:GFP)* (Figure 2G) and other specific markers of superficial cells of regular epidermis (data not shown), and displayed a different cytoplasmic composition in AFOG trichrome stainings (Figure 2F). Furthermore, immunofluorescence revealed a higher keratin content of tubercle keratinocytes compared to regular epidermis (Figure 2H). Correspondingly, expression levels of the type II keratin gene *krt8* were significantly higher in breeding tubercles than in regular epidermis (Figure 2I). Furthermore, the type I keratins *krtt1c11a* (ZFIN: zgc:136902) and *krt17* (ZFIN: zgc:92061) were exclusively expressed in lower tubercle layers, but not in regular epidermis (Figure 2J and data not shown), while expression of type II keratin *krt5* and type I cytokeratin *cki* [51] was shared by regular epidermis and lower tubercle layers, but absent in upper layers of the tubercles (Figure 2K and data not shown). Strikingly, *tgm1* [52], encoding the cross-linking enzyme transglutaminase 1 involved in cornification of the mammalian epidermis [53], was exclusively expressed in upper layers of the breeding tubercles (Figure 2L).

**Figure 3:**
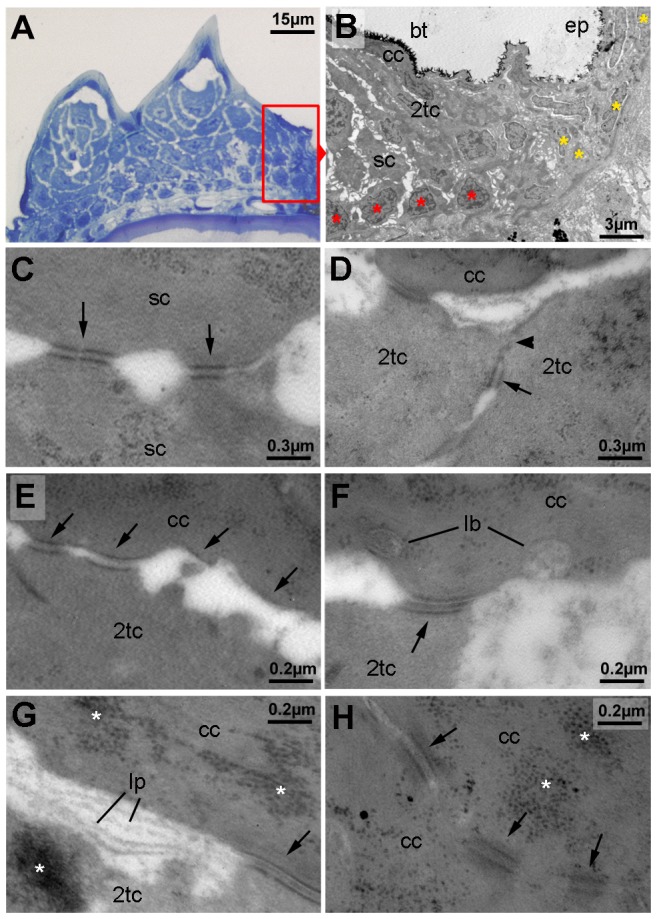
Ultrastructure of different tubercle cell layers. (A) Semi-thin section through breeding tubercles of lower jaw of a 1 year old wild-type, stained with methylene blue. For schematic overview, see also Figure 9A. Parts of the region shown in (B) are framed in red. (B–H) Transmission electron micrograph of ultrathin sections of same specimen as in (A). (B) Marginal zone of breeding tubercle. In the breeding tubercle (bt), basal cells are more regularly aligned (red asterisks) than in the adjacent epidermis (ep; yellow asterisks). Spinous (sc), second-tier (2tc) and outer cap cells (cc) are indicated. (C) Interphase between two spinous cells. Arrows point to desmosomes. (D) Interphase between two second-tier cells. At their apical side, cells are sealed to each other via tight junctions (arrowhead), directly followed by a desmosome (arrow), displaying the same spatial organization as in peridermal cells of regular epidermis (not shown) [12]. (E–G) Interphase between second-tier and outer cap cell, displaying progressive desmosomal regression (arrows) (E), extrusion of lamellar-body-like vesicles (lb) into the extracellular space (F), which is filled with material reminiscent of lipid lamellae (lp; G). Arrows point to desmosomes, asterisks mark highly abundant protein aggregates in cap and second tier cells (also in H). (H) Interphase between two outer cap cells, displaying progressive desmosomal regression and cell membrane deterioration. Abbreviations: 2tc, second-tier cell; cc, cap cell; lb, lamellar body; lp, lipid lamella; sc, spinous cell.

Striking differences were also observed at the structural level. In regular epidermis, basal and intermediary keratinocytes were of similar shapes and organized in a rather irregular pattern. In contrast, basal cells of tubercles were more regularly aligned to each other, while intermediary cells displayed a looser and spinous-like organization (Figures 2F and 3A,B), connected to each other via local desmosomes, but with wide extracellular spaces in between (Figure 3C). Tight junctions were only found in the second tier layer directly beneath the cap layer (Figure 3D), similar to their confinement to granular cells beneath the cornified layers in mammals. Desmosomes were also present between second tier and outer cap cells. However, in many samples, they were in the process of regression, leading to a partial dissociation of the two layers (Figure 3E). In addition, cap cells contained large vesicles reminiscent of lamellar bodies [54], extruding their content into the space between cap and second tier cells (Figure 3F), which was filled with material resembling the extracellular lipid lamellae in cornifying layers of the mammalian epidermis [54] (Figure 3G). Gradual loss of desmosomes was also observed between adjacent cap cells, but accompanied by cell membrane deterioration and cellular fusion (Figure 3H). Intracellular, cap and second tier cells contained large amounts of electron-dense granules (Figure 3G,H), while apart from nuclei (see below), cell organelles like mitochondria were largely absent (data not shown).

In sum, these data indicate that breeding tubercles display a more pronounced stratification than regular epidermis, consisting of different layers with distinct morphological properties and some cornification-like features in the superficial layer.

### Breeding tubercles are formed during metamorphosis and undergo regular desquamation and renewal of superficial layers

**Figure 4:**
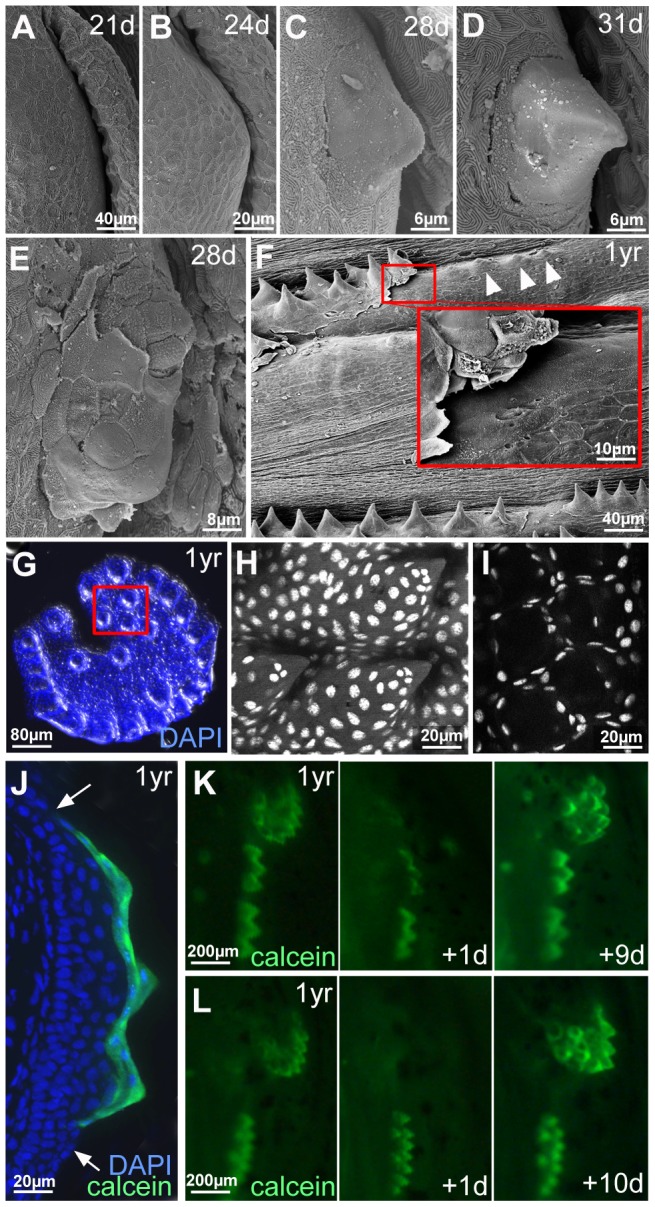
Outer cells of breeding tubercles undergo desquamation and renewal. (A–D) SEM images showing developmental time course of breeding tubercle formation on lower jaw between 21 and 31 days of development. (E) SEM image of a breeding tubercle on the lower jaw at 28 days of development. In the upper part of the image superficial cells have been lost, and former second tier cells are exposed. (F) SEM image of breeding tubercles on the pectoral fin of a male fish; 1 year of age. Inset shows magnification of boxed region. Entire rows of breeding tubercle cap layers are lifted up. Directly after desquamation of the cap layer, tubercles have a more shallow and non-spiky shape (arrowheads). (G–I) Shed cap layers of tubercles of disc-like structure on lower jaw, found in the water after spawning of 1 year old fish; stained with DAPI. Region boxed in (G) is shown in (H–I) as confocal micrographs at higher magnification; (H) maximal projection; (I) single plane. (J) Transverse cryosection through the breeding tubercles of the lower jaw of a 1 year old fish that had been incubated in calcein solution; calcein fluorescence in green; nuclei stained with DAPI in blue. Arrows point to outer layer of regular epidermis. (K,L) Calcein fluorescence of breeding tubercle region on the lower jaw of live fish, 1 year of age, repetitively stained with calcein and monitored daily. Days after first image acquisition are indicated (+1 d, +9 d, +10 d).

Regular epidermis becomes multi-layered during metamorphosis [12], with final thicknesses between 3 and 10 layers, depending on the position on the body (data not shown). Breeding tubercles develop at the same time. At 21 dpf (6.0–6.5 mm body length) and 24 dpf (6.5–7.0 mm), the future breeding tubercle domain on the lower jaw was still covered by microridge-bearing peridermal cells (Figure 4A,B). At 28 dpf (7.5–9.0 mm), first elevations with microridge-free outer cells were present (Figure 4C), which had acquired the mature spiky shape at 31 dpf (10.0–11.0 mm) (Figure 4D).

Already at early elevation stages, tubercles were observed in which some of the smooth outer cells had been lost, exposing second tier cells with a rougher surface (Figure 4E), possibly reflecting former contact points to the lost outer cells. During later stages, entire sheets of outer cells were lifted up (Figure 4F) and shed. Corresponding spiky discs (Figure 4G–I) or rows were found in the water, in particular after spawning. Confocal analyses after DAPI-staining revealed that the spikes of these sloughed structures were hollow, indicating that they consisted solely of cap layers. To investigate whether shed cap layers are renewed, we took advantage of their unique property to be readily and permissively stained with externally applied dyes like calcein (Figure 4J) or methylene blue, reflecting their loss of cell membrane integrity, a hallmark of cell death [55]. In contrast, second tier and deeper cells remained unstained. In daily analyses of the same fish over several weeks, calcein permeability of individual discs or rows was randomly lost within one day and only regained after 7–14 days (Figure 4K,L; n = 12), suggesting that shed cap cells are replaced by second tier cells and that it takes the latter 7–14 days to terminally differentiate.

### Spinous and cap layers of breeding tubercles display high levels of activated caspase 3, whereas basal layers exhibit high proliferation rates

**Figure 5:**
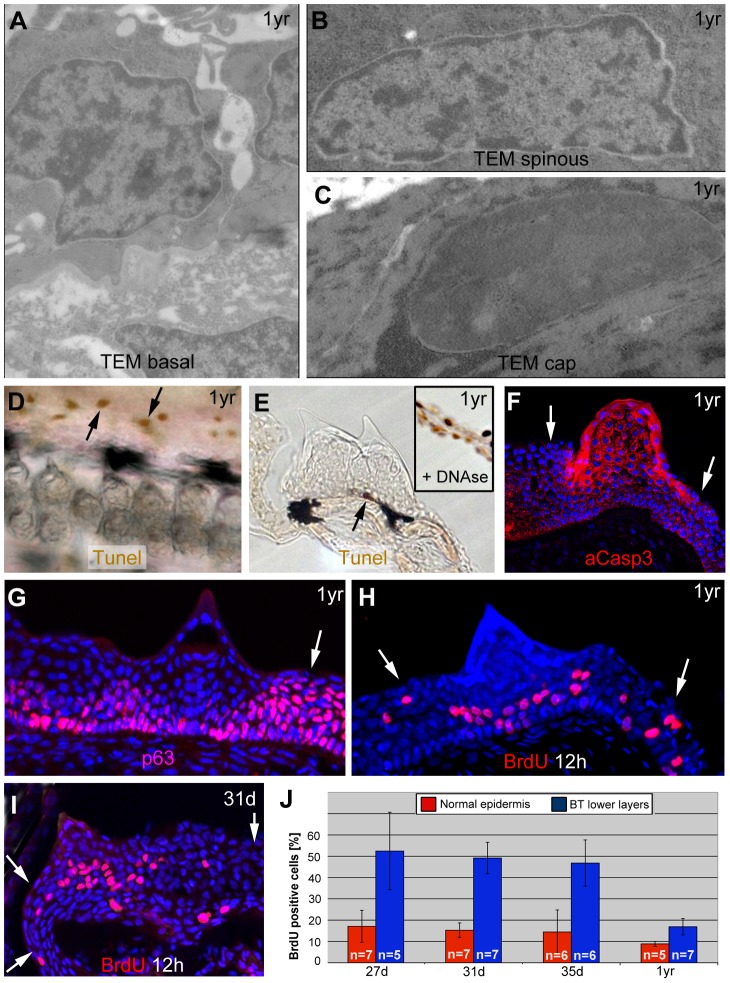
Basal tubercle cells display increased proliferation rates, upper cells increased levels of activated caspase 3 in the absence of other hallmarks of cell death. (A–C) TEM images of same specimen as shown in Figure 3, revealing nuclei with normal chromatin in basal and spinous cells of breading tubercles (A,B), whereas the chromatin of outer cap cells is more condensed (C). (D,E) TUNEL staining of pectoral fins of male fish; 1 year of age; whole mount (D) and transverse sections (E). Single TUNEL-positive cells are present in tubercle-free, inter-ray region (D, arrows) and in basal layer of breeding tubercles (E; arrow), whereas upper layers of breeding tubercles are TUNEL-negative (E). Inset in (E) shows a section through the inter-ray region after DNase treatment, with TUNEL signals in all nuclei (positive control). (F–I) anti-activated caspase 3 immunofluorescence (IF) (F), anti-p63 IF (G), anti-BrdU iIF after BrdU incorporation for 12 hours (H,I), co-stained with DAPI; transverse section through breeding tubercle of lower jaw; 1 year old fish (F–H); 31 day old fish (I). Arrows point to regular epidermis. (J) Percentages of BrdU-positive nuclei of specimens as shown in (H,I) in regular epidermis (red) and lower (BrdU-positive) layers of breeding tubercles (blue). Standard deviations and numbers of evaluated samples (n) are indicated. For all stages, differences between regular epidermis and breeding tubercles are statistically significant: 27 d, p = 0.000431; 31 d, p = 0.00000006; 35 d, p = 0.000178; 1 yr, p = 0.000295. Abbreviations: d, days; yr, year.

In contrast to mammalian corneocytes, cap cells of breeding tubercles still contain their nuclei. However, compared to the nuclei of basal and spinous keratinocytes (Figure 5A,B), their chromatin was strongly condensed (Figure 5C), similar to the pyknosis apparent during cell death [56]. Nevertheless, cap cells were TUNEL (Terminal deoxynucleotidy transferease dUTP nick end labelling)-negative (Figure 5D,E), pointing to the absence of DNA fragmentation, whereas they showed high levels of activated caspase 3 (aCasp3; Figure 5F). This cysteine protease, which does not only execute apoptosis, but also promotes differentiation of embryonic keratinocytes [39], [48], was also present in spinous cells in intermediary tubercle layers (Figure 5F), which had normal nuclei (Figure 5B) and excluded externally applied dyes (Figure 4J), thus lacking all hallmarks of cell death. In contrast, aCasp3 was absent from basal tubercle layers and regular epidermis (Figure 5F). This aCasp3 distribution pattern was largely complementary to that of p63, which was present in all layers of regular epidermis, but confined to lower layers of breeding tubercles (Figure 5G). Cell proliferation, assayed via BrdU incorporation, displayed the same pattern like p63, complementary to aCasp3 (Figure 5H). However, proliferation rates in lower tubercle layers were significantly higher than in regular epidermis, both in fully grown (Figure 5H,J) as well as in developing tubercles (Figure 5I,J).

In sum, in contrast to regular epidermis, where keratinocytes of all layers display comparable p63 and proliferation levels while lacking activated caspase 3, at least two domains can be distinguished in breeding tubercles: aCasp3-positive cells in the upper layers that are postmitotic and undergo more advanced differentiation, and aCasp3-negative keratinocytes at the base, with proliferation rates even higher than in regular epidermis. Furthermore, aCasp3 in upper layers is not correlated with apoptosis.

### TAp63 and p53 mutants display compromised breeding tubercle development

**Figure 6:**
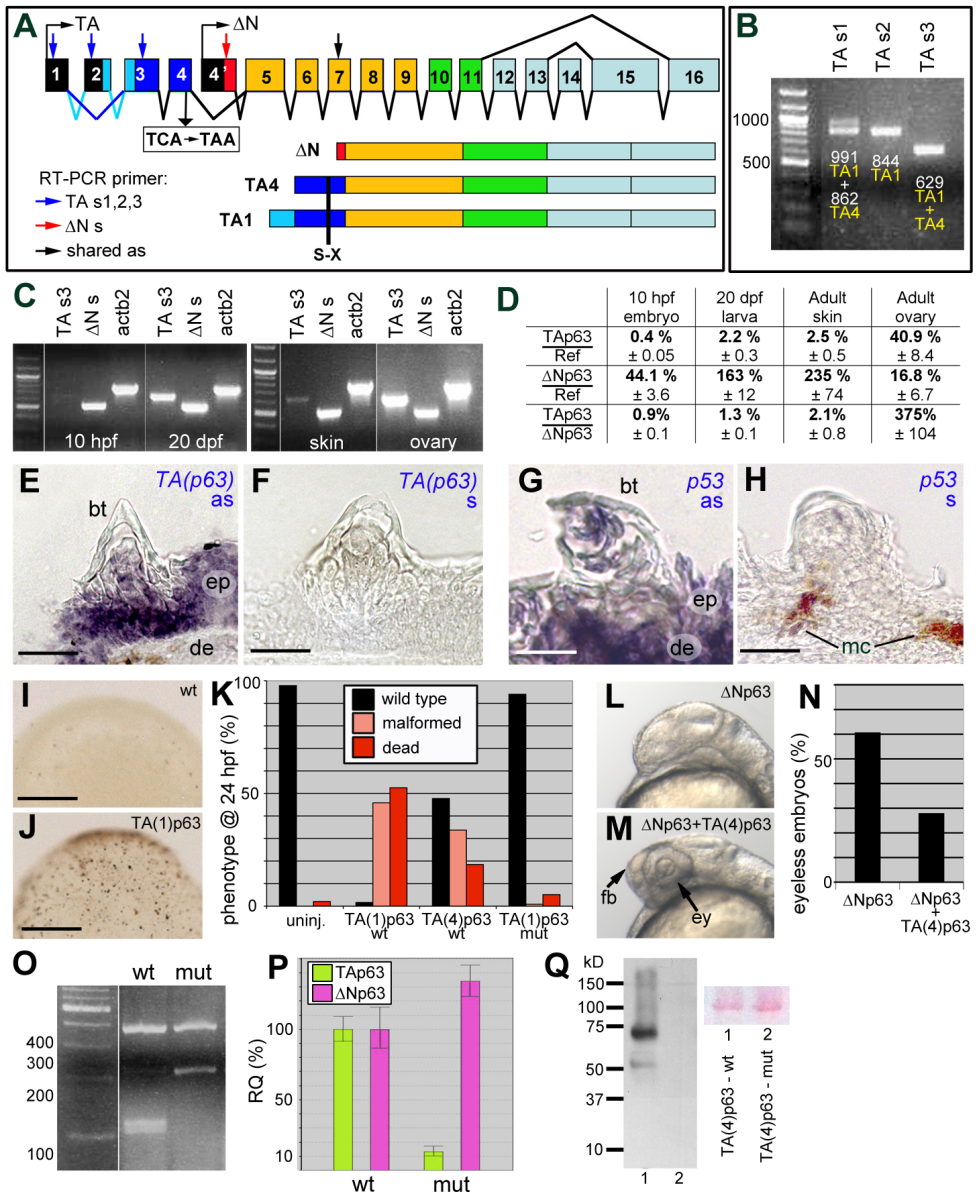
Structure, expression and biological activity of zebrafish TAp63, and molecular features of the mutant hu2525 allele. (A) Schematics of structure of zebrafish *p63* gene and of the encoded TAp63 (TA1 and TA4) and ΔNp63 isoforms. The different C-terminal isoforms (a,b,g) are not considered. The TA-specific transactivation domain is in dark (TA4) or light and dark (TA1) blue, the ΔN-specific N-terminal domain in red. Positions of primers used for RT-PCR analyses in (B–D) are indicated. Skipping of TA-specific exon 2 (indicated by dark blue lines) leads to the formation of the shorter TA4 isoform. The position of the TCA->TAA nonsense mutation (hu2525), and the resulting truncations of all TAp63 isoforms are indicated. (B) RT-PCR analyses of adult zebrafish skin, using indicated alternative TA-specific sense primers (for exact sequences and positions, see Figure S1), together with an antisense primer in exon 7 shared between TA and ΔNp63. TAs1 generates two bands, reflecting the presence or absence of exon2-encoded sequences and representing either TA1 (0.991 kb band; exon 2 present) or TA4 (0.862 kb band; exon 2 absent). TAs2 positioned in exon2 generates one band, representing TA1. TAs3 positioned in exon 3 shared by TA1 and TA4 generates one band. (C) RT-PCR analyses of TA1+TA4 and ΔNp63 expression at different stages of zebrafish development (10 hpf = end of grastrulation; 20 dpf = onset of metamorphosis) and in skin and ovaries of adult zebrafish (1 year). Identical results were obtained in three independent experiments. (D) Quantitative RT-PCR analyses of *ΔNp63* and *TAp63* transcript levels relative to standard *rps23* transcript, and ratios of *ΔNp63* and *TAp63* isoforms in whole embryos/larvae at 10 hpf and 20 dpf and in adult skin and ovaries. (E–H) in situ hybridization with TA-specific TAp63 (E,F) and p53 (G,H) antisense (E,G) and sense control (F,H) RNA probes; transverse sections through breeding tubercles of lower jaw of wild-type fish, 1 year old. Breeding tubercles (bt), regular epidermis (ep), dermis (de) and melanocytes (mc) are indicated. (I,J) Tunel staining of apoptotic cells in uninjected embryo (I) and embryo injected with *TA(1)p63γ* mRNA (J); mid-gastrula stage (8 hpf). View on animal pole. (K) Quantification of TAp63-induced phenotypes (severe malformations and death) at 24 hpf, resulting from apoptosis during mid-gastrula stages, as shown in panels I,J (ratio between affected and total (n) embryos; in %; 3 independent injections). TA(1)p63γ(wt), n = 240; TA(4)p63γ(wt), n = 314; TA(1)p63γ(hu2525 mut), n = 234. (L,M) Representative examples of embryos at 32 hpf, injected with ΔNp63α1 mRNA (L) or co-injected with ΔNp63α1 and TA(4)p63γ mRNA (M); lateral views on head region. Over-expression of ΔNp63 leads to the loss of forebrain (fb) and eyes (ey), resulting from ventral shifts during early patterning of the embryonic ectoderm (L) [58], while these structures are rescued in the embryo over-expressing both ΔNp63 and TAp63 (M). (N) Quantification of headless phenotypes (in % of wild-type plus headless embryos (n); 3 independent injections; ΔNp63α1, n = 277; ΔNp63α1+TA(4)p63γ(wt), n = 354). For simplicity, dead and malformed embryos in the co-injection are not considered. However, frequencies were significantly lower (25%) compared to embryos only injected with TA(4)p63γ(wt) (52%; see panel K), indicating that ΔNp63 also alleviates the pro-apoptotic effect of TAp63. (O) *Mbo*I RFLP analysis of TAp63 cDNA fragment amplified from skin of adult wild-type or *TAp63^hu2525/hu2525^* mutant fish. The mutant only contains transcripts harbouring the hu2525 mutation that destroys an *Mbo*I site (GATC; see Figure S3), resulting in a 208 bp instead of the two wild-type cleavage products of 101 bp and 107 bp. (P) Quantitative RT-PCR analyses of TAp63 and ΔNp63 mRNA levels in adult skin of wild-type and *TAp63^hu2525/hu2525^* mutant fish (2 independent experiments), revealing that in the mutant, TAp63 levels are reduced to 13.3%, and ΔNp63 are elevated to 134% of the wild-type levels. (Q) Anti-Myc-tag Western blot of lysates from embryos injected with mRNA encoding wild-type (lane 1) or hu2525 mutant N-terminally Myc-tagged zebrafish TA(4)p63γ. Ponceau red staining of membrane is shown as loading control. Calculated protein masses of 6xMyc-TA(4)p63γ proteins are: wt, 65.3 kDa; mut, 14.4 kDa. Identical results were obtained in 3 independent experiments. Scale bars are: 20 µm (E–H), 200 µm (I,J).

In light of the described observations, we next studied breeding tubercle development in zebrafish loss-of-function mutants in p53 and TAp63, potential regulators of keratinocyte differentiation and proliferation in mammalian cell culture systems [29], [31]–[33]. The *p53^zdf1^* allele bears an (M214K) exchange of a conserved amino acid residue in the DNA-binding domain that compromises p53 activity [57]. For zebrafish *p63*, only ΔN isoforms had been described thus far [58], [59]. However, via exon prediction of genomic sequences upstream of the ΔNp63-specific exon (Ensembl accession number ENSDARG00000044356) and validation via RT-PCR analyses and cDNA sequencing, two N-terminal TAp63 isoforms, TA1 and TA4, were identified (Figures 6A and S1) that are similar to the corresponding isoforms in mammals [25] (39% aa identity; 55% aa similarity; Figure S2A,B; GenBank accession numbers KF682365, KF682366). TA1 corresponds to mammalian full-length TAp63, TA4 to Δ40TAp63, which was the initially described human isoform [18]. Furthermore, the exon-intron organization is conserved between mammals and fish (Figure S1), and sequences from shark to human segregate in the expected phylogenetic pattern (Figure S2C).

Both predicted TAp63 isoforms (TA1, TA4) were expressed in adult zebrafish skin (Figure 6B). Comparative regular (Figure 6C) and quantitative real-time RT-PCR (Figure 6D) analyses further revealed almost exclusive presence of ΔNp63 transcripts in early zebrafish embryos, similar to the situation in mouse [60]. At the onset of metamorphosis (20 dpf), TAp63 levels were approximately 5fold increased, but still constituted only 1.3% of the total p63 levels (Figure 6D). Also in adult zebrafish skin, TAp63 was expressed at much lower levels than ΔNp63 (approx. 2%), whereas higher TAp63 transcript levels were found in the ovary, the site of essential TAp63 function in mouse [34] (Figure 6D). In situ hybridizations with an isoform-specific RNA probe further revealed that TAp63 was expressed in regular epidermis as well as in all layers of the breeding tubercles (Figure 6E,F), similar to the expression pattern of p53 (Figure 6G,H).

To investigate whether the zebrafish TAp63 transcripts are translated into biologically functional proteins, we carried out over-expression studies in zebrafish embryos. Injected synthetic mRNA encoding the TA1 or TA4 isoform of zebrafish TAp63γ caused widespread apoptosis during gastrulation stages (Figure 6I,J), leading to embryonic death or severe malformations during further development (Figure 6K), similar to the previously reported effects of injected zebrafish p53 mRNA [61]. In addition, zebrafish TAp63γ mRNA significantly rescued the (headless) phenotype caused by ΔNp63α over-expression (Figure 6L–N), as previously reported for mouse TAp63 and p53 [58]. This indicates that zebrafish TAp63 transcripts give rise to a protein with p53-like pro-apoptotic and ΔNp63-antagonizing activities.

Using target-selected mutagenesis [62], a TA-specific zebrafish p63 mutant (*Tp63^hu2525^*) mutant was isolated that bears a TCA->TAA nonsense mutation (S48X) in the last TA-specific exon truncating the N-terminal isoforms of TAp63, while leaving ΔNp63 isoforms unaffected (Figures 6A and S3). RT-PCR followed by restriction fragment length polymorphism (RFLP) analysis of skin samples confirmed the presence of the premature stop codon in *TAp63* transcripts of *TAp63^hu2525/hu2525^* animals (Figure 6O). Furthermore, qRT-PCR revealed that in *TAp63^hu2525/hu2525^* mutant skin, TAp63 mRNA levels were more than 7.5 fold reduced compared to wild-type siblings, whereas ΔNp63 mRNA levels were slightly up-regulated (Figure 6P), suggesting that the mutant TAp63 transcripts undergo nonsense-mediated mRNA decay and that TAp63 normally has a subtle negative effect on ΔNp63 transcription. Unfortunately, we could not investigate TAp63 proteins in *TAp63^hu2525/hu2525^* mutants, as all tested p63 antibodies failed to detect p63 proteins after Western blotting of zebrafish extracts (data not shown). However, upon injection of synthetic mRNAs encoding N- or C-terminally Myc-tagged zebrafish TAp63γ, wild-type mRNA gave rise to full-length fusion protein, whereas hu2525 mutant mRNA yielded no products (Figure 6Q and data not shown), suggesting that the stop codon cannot be by-passed, that no internal start site is used, and that the truncated protein is unstable. Furthermore, in contrast to wild-type TAp63γ, hu2525 mutant mRNA lacked pro-apoptotic activity upon over-expression in early zebrafish embryos (Figure 6K; compare columns 2 and 4). Together, this suggests that the hu2525 mutant is a TAp63 null.

**Figure 7:**
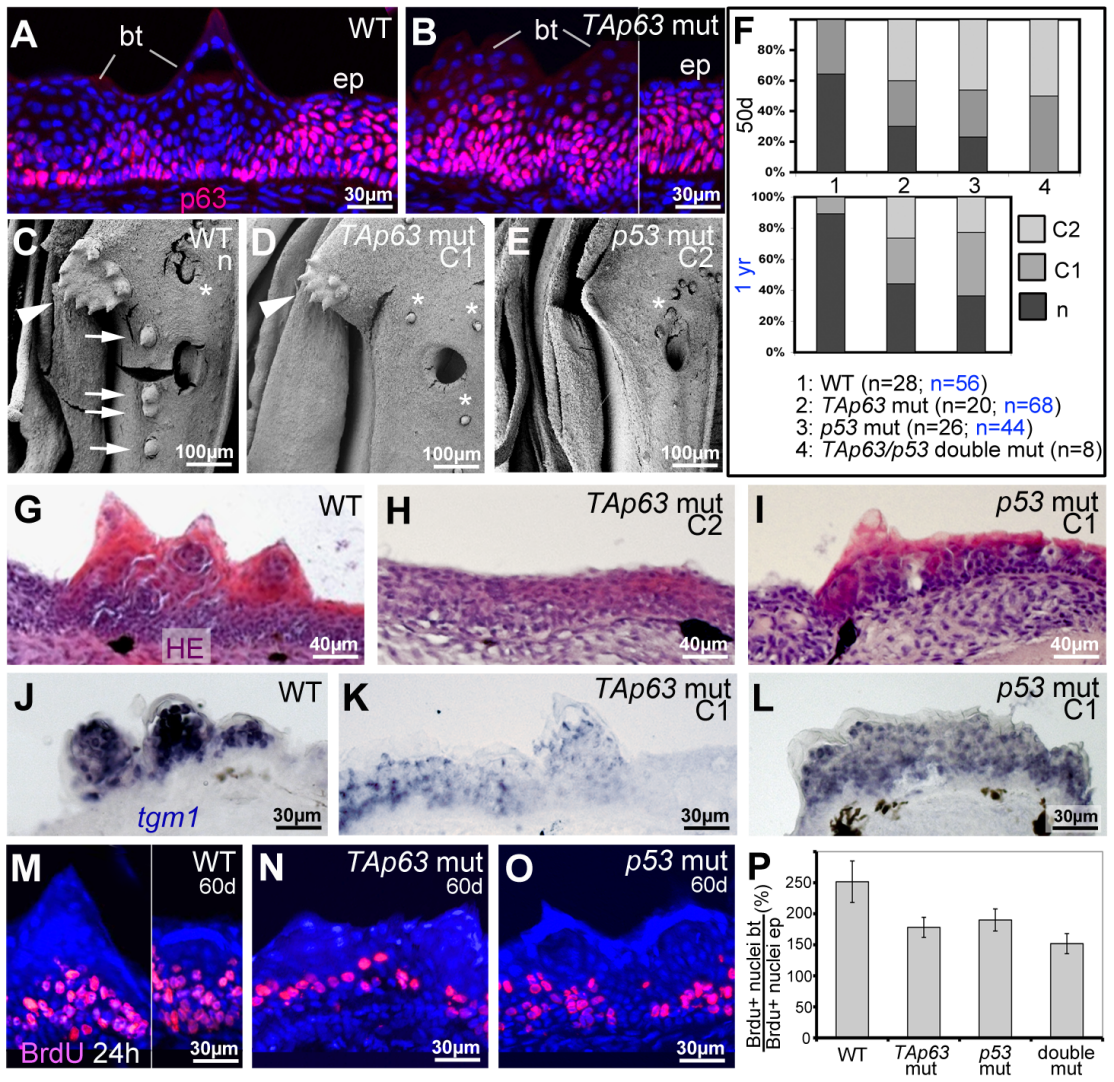
TAp63 and p53 mutants display reduced proliferation in basal layers and reduced differentiation in upper layers of tubercle remnants. (A,B) anti-p63 immunofluorescence; transverse sections through lower jaw of wild-type (A) and TAp63 mutant (B), 1 year of age. Breeding tubercles (bt) and adjacent regular epidermis (ep) are indicated. For larger field images and p53 mutant, see Figure S4E–G. (C,D,E) SEM images of lower jaw, ventral view. The TAp63 mutant (D; intermediate phenotype; C1) has a smaller disc (arrowheads in C,D) with smaller and fewer tubercles, while the posterior row of tubercles (arrows in C) is missing. The p53 mutant (E; strong phenotype; C2) lacks all breeding tubercles. Asterisks mark smaller, more randomly distributed appendages, which most likely are unculi [85] that are unaffected in the mutants. (F) Distribution of phenotypic strengths (n, normal; C1, intermediate; C2, strong) in lower jaw tubercles of wild-type, TAp63 mutant, p53 mutant and TAp63/p53 double mutant fish at an age of 50 days (upper panel) and 1 year (lower panel), determined via calcein staining (for example, see Figure 8V,X). Numbers of evaluated specimens are indicated. Similar results were obtained for the pectoral fins of males (not shown). (G–I) Hematoxylin & eosin staining; transverse sections through breeding tubercle disc region of lower jaw of wild-type (G), TAp63 mutant (H; strong phenotype; C2), and p53 mutant (I; intermediate phenotype; C1) fish at 1 year. For larger field images, see Figure S4A–D. (J–L) *tgm1 in situ* hybridization; transverse sections through breeding tubercle disc region of lower jaw at an age 1 year. In TAp63 mutant (K), *tgm1* levels in upper layers of breeding tubercles are strongly reduced compared to wild type (J), whereas in the p53 mutant (L), the effect is weaker. Identical results were obtained in three independent experiments. (M–O) anti-BrdU immunofluorescence, combined with nuclear DAPI staining; transverse sections through breeding tubercles on the lower jaw of wild-type (M), TAp63 mutant (N), and p53 mutant (O) fish at an age of 60 days and directly after incubation with BrdU for 24 hours. (P) Ratios between keratinocyte proliferation rates in lower layers of breeding tubercles and regular epidermis (epidermal rates were unaltered in all cases; BrdU+ nuclei_bt_/total nuclei_bt_//BrdU+ nuclei_ep_/total nuclei_ep_; in %). Standard deviations are indicated. Numbers of evaluated samples were: WT, 36 sections from 4 fish; TAp63 mut, 28 sections from 4 fish; p53 mutant, 31 sections from 4 fish; TAp63/p53 double mutant, 21 sections from 3 fish. Observed decreases in keratinocyte proliferation in tubercles of mutants are statistically significant (Student's t-test; WT - TAp63 mut, p = 0.0041; WT - p53 mut, p = 0.008; WT – TAp63/p53 double mut, p = 0.0033).

Like *p53^zdf1/zdf1^* mutants, *TAp63^hu2525/hu2525^* mutants were viable and fertile. Anti-p63 immunofluorescence analysis revealed normal staining in regular epidermis of TAp63 mutants (Figure 7A,B; Figure S4E–G), suggesting that ΔNp63 is the major p63 isoform of the adult zebrafish skin, consistent with our RT-PCR data. However, both TAp63 and p53 mutants displayed specific breeding tubercle deficiencies of variable strength (C1,C2). SEM (Figure 7C–E), calcein stainings (Figure 7F) and histological sections (Figure 7G–I; Figure S4A–D) revealed complete absence (C2) in some individuals and reduced numbers and/or sizes of tubercles (C1) in others, while frequencies of strongest phenotypes were significantly increased in TAp63/p53 double mutants (Figure 7F). At the molecular level, even tubercle remnants of more weakly affected (C1) TAp63 and/or p53 mutants displayed significant alterations compared to wild-type tubercles, such as reduced *tgm1* (Figure 7J–L) and ectopic (ΔN)p63 expression (Figure 7A,B) in upper layers, as well as reduced proliferation rates at the base, which again was most prominent in TAp63/p53 double mutants (Figure 7M–P). In sum, this makes the molecular signature of breeding tubercle remnants of weaker TAp63/p53 mutants more similar to that of the regular epidermis.

Together, our data suggest that TAp63 and p53 are required for proper keratinocyte proliferation at the base of breeding tubercles, as well as for cornification-like differentiation processes in upper layers.

### TAp63 and p53 act via Notch signalling and caspase 3

**Figure 8:**
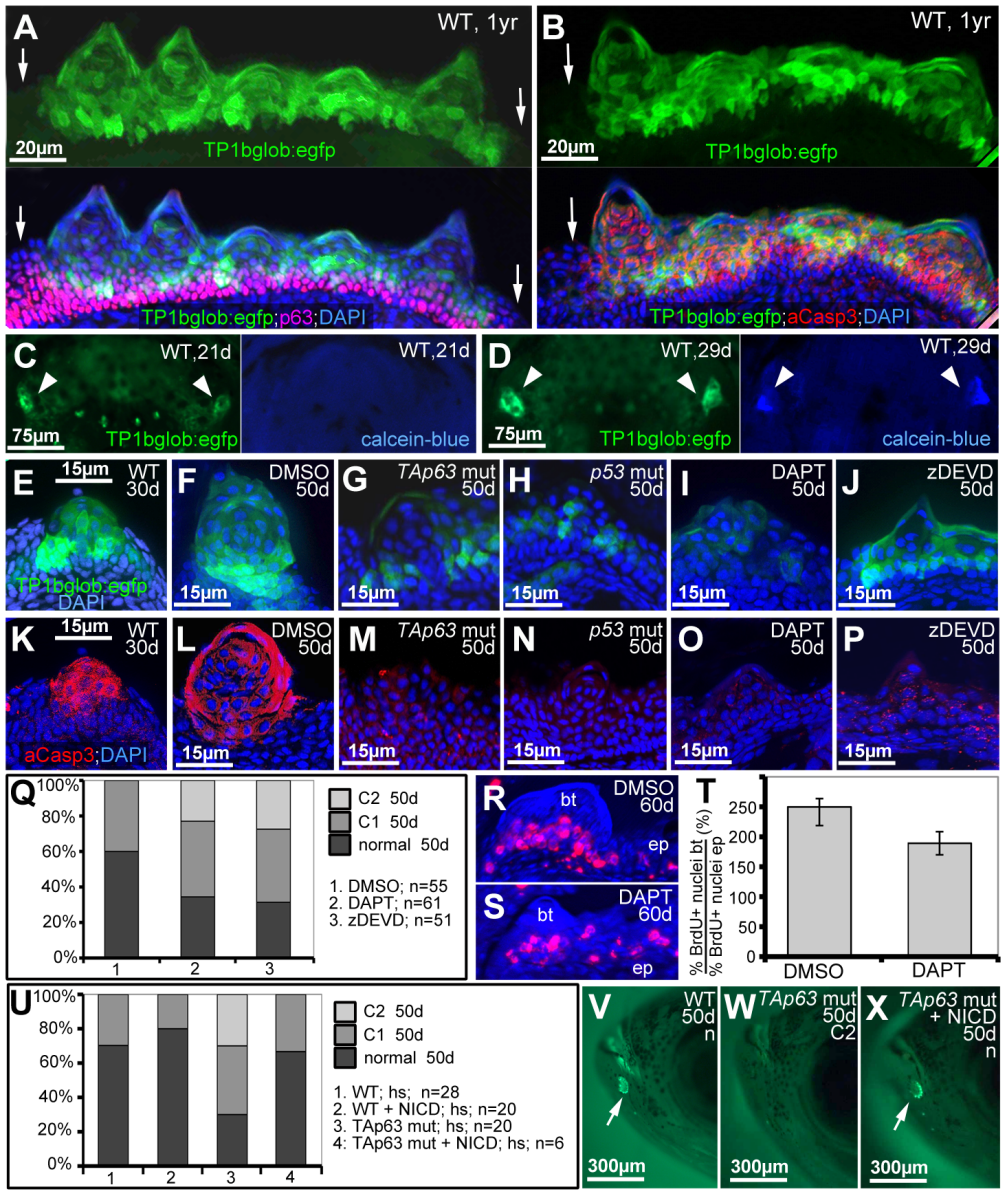
TAp63 and p53 act via Notch signalling and activated caspase 3. (A,B) Double anti-p63 (in red) and anti-GFP (in green) (A) or anti-aCasp3 (in red) and anti-GFP (in green) (B) immunofluorescences of Notch signalling reporter fish at 1 year of age; transverse sections through breeding tubercles in the disc region of the lower jaw; green channel in upper panels; merged images including DAPI staining in lower panels. Regular epidermis is indicated by arrows. (C,D) GFP and calcein-blue *in vivo* imaging of lower jaw of Notch signalling reporter fish at 21 dpf (C) and 28 dpf (D); ventral views; green channel to the left, blue channel to the right; the bilateral breeding tubercle disc regions are indicated by arrowheads. (E–P) GFP in vivo imaging of Notch signalling reporter (in green; E–J) and anti-aCasp3 immunofluorescence (in red; K–P) in untreated controls at 30 days (E,K), DMSO-treated controls (F,L), TAp63 mutants (G,M) p53 mutants (H,N), DAPT-treated wild types (I,O) or zDEVD-treated wild types (J,P), all at 50 days of age; transverse sections through breeding tubercles in disc region of lower jaw. For each condition, identical results were obtained in all of at least 10 investigated individuals (see Figure S5). (Q) Distribution of phenotypic strengths (normal; intermediate, C1; strong, C2) in lower jaw tubercles of DMSO-, DAPT- or zDEVD-treated wild-type fish; all fish (numbers indicated; n) were evaluated at 50 days of age after calcein staining (not shown). (R,S) anti-BrdU immunofluorescence, combined with nuclear DAPI staining; transverse sections through breeding tubercles on the lower jaw of DAPT- (M), and DMSO-treated control fish (N) at an age of 60 days and directly after incubation with BrdU for 24 hours. Scale is as in panels (E–P). (T) Ratios between keratinocyte proliferation rates in lower layers of breeding tubercles and regular epidermis (epidermal rates were identical; BrdU+ nuclei_bt_/total nuclei_bt_//BrdU+ nuclei_ep_/total nuclei_ep_; in %). Standard deviations are indicated. Numbers of evaluated samples were: DMSO, 30 sections from 5 fish; DAPT, 19 sections from 4 fish. Difference is statistically significant (Student's t-test; p = 0.0008). (U) Distribution of phenotypic strengths (normal; intermediate, C1; strong, C2) in lower jaw tubercles of TAp63 mutant and wild-type siblings versus *Tg(5xUAS-E1b:6xMYC-notch1a); Tg(-1.5hsp70l:Gal4)* double transgenic TAp63 mutants or wild-type siblings after heatshock-treatments from 20–50 days of development; all fish (numbers indicated; n) were evaluated at 50 days of age after calcein staining (see V–X for examples). (V–X) 50 day old non-transgenic control (WT; V), non-transgenic TAp63 mutants (W) and double transgenic TAp63 mutant after transgenic NICD expression from 20–50 days of development (X); calcein in vivo staining, lateral views on head. Arrows in (V,X) point to stained breeding tubercles in disc region of lower jaw. Abbreviations: ep, regular epidermis; bt, breeding tubercle.

In light of their known roles in keratinocyte differentiation in mouse [39], we also explored the involvement of Notch signalling and caspase 3 during zebrafish tubercle development, as well as their epistatic relationships to TAp63/p53. Differentiating tubercle keratinocytes displayed strong Notch signalling, as revealed by *Tg(TP1bglob:eGFP)^um13^*, a transgenic in vivo reporter with 12 RBP-J_k_ binding sites [63], which was strongly expressed in upper layers of breeding tubercles, but not in lower layers and regular epidermis, complementary to the distribution of (ΔN)p63 (Figure 8A), but overlapping with aCasp3 (Figure 8B). Double labelling with calcein blue as a marker of differentiated cap cells revealed that during development, Notch signalling in the tubercle domain is initiated several days before the first cap cells have differentiated (Figure 8C,D). Furthermore, TAp63 and p53 mutants displayed strongly reduced Notch signalling and aCasp3 levels in their (significantly smaller) tubercles at 50 dpf (Figure 8F–H,L–N), although wild-type tubercles of similar sizes at 30 dpf were strongly positive for both (Figure 8E,K).

To study whether Notch signalling and caspase 3 are required for breeding tubercle formation, we treated wild-type fish from 20 to 50 dpf with the specific Notch/g-secretase inhibitor DAPT or the caspase 3 peptide inhibitor zDEVD-fmk. While both treatments did not affect the general conditions and growth of the fish (Figure S5), DAPT-treatment caused significant reductions in the numbers and sizes of tubercles, in the activity of the Notch reporter and in aCasp3 levels (Figure 8I,O,Q), as also seen in p53 and TAp63 mutants (Figures 7F and 8G,H,M,N). zDEVD-fmk treatment had similar effects on tubercle numbers and sizes and on aCasp3 levels (Figure 8P,Q), while leaving Notch signalling unaffected (Figure 8J). Furthermore, despite the restriction of Notch signalling to upper breeding tubercle layers (see above), DAPT treatment led to a significant reduction of keratinocyte proliferation at the base of the tubercles (Figure 8R–T), comparable to the effects caused by loss of TAp63 and p53 function (Figure 7P). Finally, we re-introduced Notch signalling into TAp63 mutants, using a binary transgenic approach for temporally controlled expression of the constitutively active intracellular domain of Notch1 (NICD) [64]. While heatshock-induced NICD expression from 20–50 dpf had little effect in wild-type fish, it significantly elevated tubercle numbers in TAp63 mutants back to wild-type conditions (Figure 8U–X).

Together, these data suggest that TAp63/p53, Notch signalling and aCasp3 constitute a linear pathway required for proper breeding tubercle formation.

## Discussion

Both the *in vivo* role of TAp63 isoforms during vertebrate keratinocyte development and the stratification status of the epidermis of adult zebrafish were not fully understood. Here, we present data shedding new light onto both questions, identifying the zebrafish breeding tubercles as epidermal appendages with more advanced stratification and keratinocyte differentiation, and with regular desquamation and self-renewal, while presenting genetic evidence for an *in vivo* involvement of a TAp63/p53->Notch->caspase 3 pathway in these processes.

### Breeding tubercles as sites of more advanced keratinocyte differentiation and regular self-renewal

Thus far, it had been unclear whether the epidermis of adult zebrafish undergoes self-renewal as in mammals. Our transgenic lineage analyses indicate that both in regular epidermis and in breeding tubercles, derivatives of basal keratinocytes can be found in the outer cell layer (Figure 1). Furthermore, long-term observations of individual fish show that outer layers of breeding tubercles are shed off, and that it takes cells of the next layer several days to fully develop outer layer properties before they undergo desquamation themselves (Figure 4).

Electron microscopy and marker analyses further revealed striking differences in the differentiation of keratinocytes in breeding tubercles versus regular epidermis. In regular epidermis, basal and intermediary cells display largely identical ultrastructural features [12]. In addition, they are (ΔN)p63-positive and mitotically active throughout all layers, and express the same keratin genes (Figures 2 and 5). Only when ending up in the superficial layer do they become strikingly different, express specific keratins and other structural proteins, including components of tight junctions, which are only present in this layer. Strikingly, basal keratinocyte-derived superficial cells of the regular epidermis are indistinguishable from persisting cells of the early embryonic enveloping layer, with which they form a uniform and continuous periderm-like sheet (Figure 1E).

**Figure 9:**
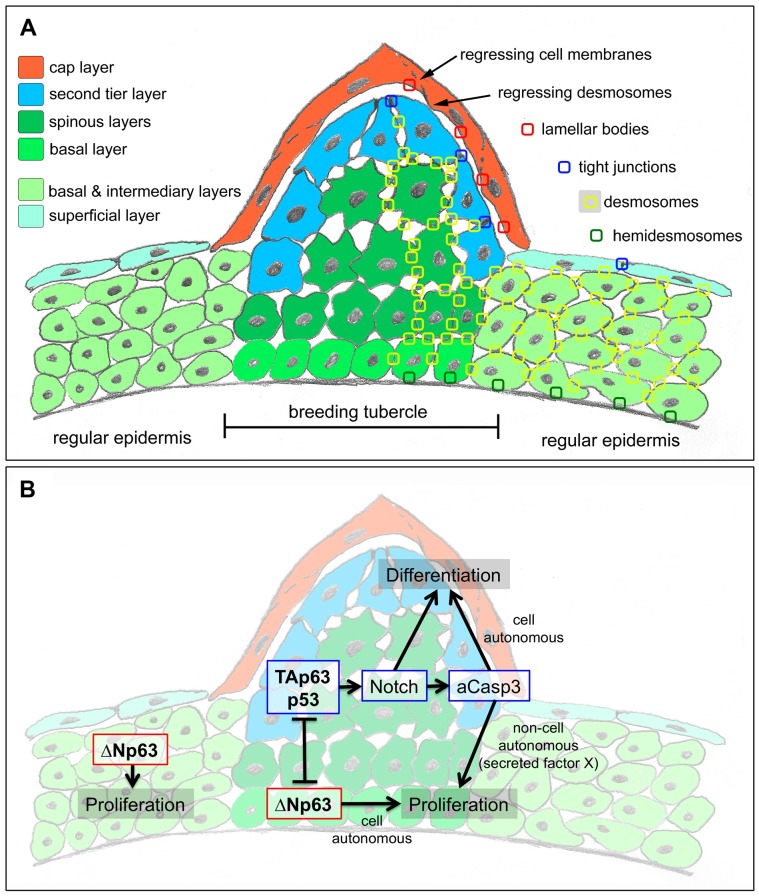
Schematics of breeding tubercle organization and regulation. (A) Schematic of cellular composition of breeding tubercles and regular epidermis. The different layers and cell types are colour-coded. On the right side, the sites of cellular structures and junctions shown in Figure 3 (TEM) are indicated. (B) Model for the dual role of TAp63/p53 on keratinocyte proliferation and differentiation, and its connection to ΔNp63. For details, see text.

In contrast, breeding tubercles exhibit a much more pronounced stratification (see Figure 9A for schematic drawing). They consist of more epidermal cell layers and display a keratin gene expression pattern strikingly different from that of regular epidermis (Figure 2). Cell proliferation is confined to basal layers, while the expression of transglutaminase 1, a crucial cross-linking enzyme during mammalian cornification [53], which is absent in regular zebrafish epidermis, is confined to upper layers (Figures 2 and 5). Structurally, at least 4 different layer types can be distinguished. The basal layer, in which cells are organized in a much more regular and columnar fashion than in regular epidermis, several spinous layers, a second-tier layer, in which cells are sealed to each other via tight junctions, and a heavily keratinized outer cap layer (Figures 3 and 9A). This organization is similar to that of the epidermis of adult mammals. In addition, the presence of extruding lamellar body-like vesicles and of lipid deposits at the second tier – cap layer interphase is reminiscent of lipid envelope formation during mammalian cornification [54], while the progressive degradation of desmosomes between second tier and cap layer cells resembles the fate of corneodesomosomes in mammalian corneocytes [65]. However, there are also differences between zebrafish breeding tubercles and the cornifying mammalian epidermis. Thus, despite their high keratin content, tubercle keratinocytes lack obvious intermediary filament bundles and a cornified envelope (CE), in line with the absence of genes encoding the keratin-bundling protein filaggrin and the CE components loricrin and involucrin in teleost genomes [66]. Furthermore, in contrast to mammalian corneocytes, sloughed cap cells of tubercles still contain their nuclei and display some (loss of cell membrane integrity, nuclear pyknosis), but not all hallmarks of apoptosis [55], [56] (Figures 4 and 5).

Overall, this makes differentiated zebrafish tubercle keratinocytes more similar to the “immature horny cells with nuclei” observed in fetal human epidermis, which are supposed to represent a “transition phase of keratinisation” [2]. In line with such a more basal state, keratinocyte differentiation in zebrafish tubercles requires Notch signalling and caspase 3, regulators that are also needed for early steps of epidermal cornification in mammals [37], [39].

### A linear TAp63->Notch->caspase 3 pathway drives advanced keratinocyte development in breeding tubercles

There is compelling evidence in mouse and zebrafish that ΔNp63 is required during early steps of epidermal development, promoting the proliferation and stemness of basal keratinocytes, while possibly blocking differentiation processes [26], [58], [59]. However, the *in vivo* role of its counterpart TAp63 during keratinocyte development and differentiation remained elusive. Also, to our knowledge, no defects during regular keratinocyte development have been reported for p53 mutants as yet [31], [32].

Here, we revealed specific defects during keratinocyte development in breeding tubercles of zebrafish p53 and TA-specific TAp63 mutants. The used p53 allele, zdf1 (also called e7), bears a missense mutation in the DNA binding domain. Although the strongest of all available p53 mutants [57], [67], it might not be a complete functional null. However, inferences with other members of the p53/63/73 family seem very unlikely, as zdf1 homozygous embryos lack the phenotypes caused by loss of p63 or p73 [58], [59], [61], [67]. Antimorphic effects are also unlikely for the used TAp63 allele, hu2525, as the mRNA and the resulting truncated protein seem to be unstable (Figure 6P,Q). Furthermore, the protein would only contain part of the transactivating domain, while lacking DNA binding and oligomerization domains (Figure 6A). Therefore, we conclude that both TAp63 and p53 are per se essential for normal breeding tubercle development.

Similar tubercle defects as in TAp63 and p53 mutants were obtained upon chemical inhibition of Notch signalling or caspase 3 activity (Figure 8). aCasp3 levels are strongly reduced in TAp63 and p53 mutants, as well as after loss of Notch signalling, whereas Notch signalling is only lost in TAp63 and p53 mutants, but not after inhibition of caspase 3. In addition, re-introduction of Notch signalling into TAp63 mutants rescues their tubercle deficiencies. Together with their shared expression in differentiating keratinocytes of wild-type tubercles (Figures 6E,G and 8B), this provides *in vivo* evidence for the presence and requirement of a linear TAp63/p53->Notch->caspase 3 pathway.

Future studies have to elucidate the genetic control of breeding tubercle formation upstream of TAp63 and p53. As previously suggested [21], we believe that TAp63/p53 activity in zebrafish tubercles is indirectly promoted via negative interferences with its antagonist ΔNp63, which in skin is present in vast excess over TAp63 (Figure 6D), but, in contrast to TAp63 (Figure 6E), most likely restricted to the base of breeding tubercles (Figure 7A). This initial inhibition of ΔNp63 production in upper layers of the tubercle anlage might be reinforced by various negative feedbacks between TAp63/Notch/caspase and ΔNp63. Thus, in mammalian epidermal keratinocytes, Notch1 has been shown to repress ΔNp63 expression [43], while TAp63 induces caspase-dependent ΔNp63 degradation [68], consistent with the observed increased ΔNp63 transcript levels in the skin (Figure 6P) and the presence of ectopic ΔNp63 protein in upper tubercle layers of TAp63 mutant zebrafish (Figure 7A,B).

### The dual effect of TAp63->Notch->Caspase 3 on keratinocyte proliferation and differentiation

It might appear paradoxical that in addition to reduced keratinocyte differentiation in upper layers of breeding tubercles (Figure 7J–L), loss of TAp63 or p53 function also leads to reduced keratinocyte proliferation at the base of the tubercles (Figure 7M–P), pointing to both differentiation- and proliferation-promoting effects of these regulators. Reduced tubercle growth and basal keratinocyte proliferation was also obtained upon inhibition of the TAp63/p53 mediators Notch or caspase 3 (Figure 8E–T), although both are only active in post-mitotic keratinocytes in upper tubercle layers (Figure 8A,B), pointing to non-cell autonomous mitogenic effects. Interestingly, in regenerating wings of the fruitfly *Drosophila melanogaster*, the enzymatic activity of the caspase Dronc generated in apoptotic cells does not only execute cell death in a cell-autonomous manner, but also promotes proliferation of adjacent cells even when Dronc-positive cells are prevented from dying, pointing to the presence of a Dronc-dependent mitogenic signal that acts in an apoptosis-independent and non-cell autonomous manner [48], [69]. It is tempting to speculate that a similar mechanism might be at play in breeding tubercles, in which a TAp63/p53->Notch->caspase 3 pathway active in upper epidermal layers, while cell-autonomously promoting terminal keratinocyte differentiation, enhances proliferation of cells in lower layers in a paracrine fashion (via an unknown secreted factor X; Figure 9B), accounting for proper tubercle growth during development and for proper balancing between cell loss via desquamation and cell renewal during tissue homeostasis [70]. Such a proliferation-stimulating role seems in contrast to the known functions of Notch and p53 as tumour suppressors [71], [72]. However, it is in line with the initial identification of p53 as an oncogene [73] and its more recently described function in the context of metabolic control [74].

### Why TAp63 mouse mutants might lack overt defects during epidermal development

The breeding tubercle phenotype of zebrafish TAp63 and p53 mutants is not fully penetrant and variable in strength. Interestingly, however, phenotypic penetrance and average strength are significantly higher in TAp63/p53 double mutants, suggesting that the two structurally related transcription factors play partially redundant roles. Although other reasons cannot be ruled out, this suggests that a similar, possibly even more pronounced functional redundancy might also account for the apparent absence of epidermal defects in TA-specific p63 mutant mice [34]–[36]. Of note, we even identified a few TAp63/p53 double mutant zebrafish in which breeding tubercles were not completely lost. This could be due to some remaining p53 activity (see above). In addition, it might point to the existence of further partially redundant factors. TAp73, the third member of the family, is a candidate, which in mouse is expressed in all examined tissues and required side by side with TAp63 in oocytes to prevent genomic instability and female infertility [24], [34], [75]. Particular ΔNp63 isoforms might also be involved. Although in most cases they have dominant negative effects on TAp63 isoforms and p53, acting as transcriptional repressors, cases have been reported where they transactivate target genes [27] and positively cooperate with p53 [29].

Together, this demonstrates the complexity of the p53/p63/p73 system of transcriptional regulation, while revealing that it can be helpful to perform genetic analyses in different *in vivo* model systems, taking advantage of variations that have occurred during vertebrate evolution.

## Materials and Methods

### Zebrafish lines and genotyping

Unless stated otherwise, wild-type fish from a mixture of TL and EK were used. The mutant *Tp63^hu2525^* (S48X) line was generated upon our request in the Hubrecht Institute, NL, using target-selected mutagenesis (TILLING) [62]. The stable transgenic lines *Tg(krt4:creERt2)^fr33^*, *Tg(krt19:dTomato)^fr34^* and *Tg(krt19:creERt2)^fr35^* were generated using the Tol2 kit [76], [77] with the described *krt4* [49] or *krttc19e* [50] (here abbreviated as *krt19*) promoter fragments for construct generation, followed by standard injection and screening procedures.

The mutant line *Tp53^zdf1^* (M214K) [57] and the transgenic lines *Tg(krt4:GFP)^gz7^* [49], *Tg(actb2:loxP-STOP-loxP-dsREDEx)^sd5^* [78], *Tg(TP1bglob:eGFP)^um13^* [63], *Tg(5xUAS-E1b:6xMYC-notch1a)^kca3^* [64] *and Tg(-1.5hsp70l:Gal4)^kca4^* [63] have been previously described. For NICD expression, *Tg(5xUAS-E1b:6xMYC-notch1a)*, *Tg(-1.5hsp70l:Gal4)^kca4^* double transgenic fish were heat-shocked from 20–50 dpf once a day for 1 hour at 40°C.

The *TAp63^hu2525^* allele was genotyped using the dCAPS (derived Cleaved Amplified Polymorphism Sequence) method [79] with PCR primers CTGACCCCGAGGTTGTCTAA (sense) and TGCTAATCTGTATAGTATTGGAAGCT (antisense) and subsequent *Hind*III digest. The *Tp53^zdf1^* allele was identified via an RFLP (Restriction fragment length polymorphism) genotyping assay with PCR primers CCAGAGTATGTTCTGTCCA (sense) and TGATTGTGAGGATGGGCCTGCGGAATC (antisense) and subsequent *Bst*yI restriction digest.

Fish carrying the *Tg(-1.5hsp70l:Gal4)^kca4^* or *Tg(5xUAS-E1b:6xMYC-notch1a)^kca3^* transgene were identified by PCR transgene amplification with the primers CGGGCATTTTACTTTTATGTTGC (gal4, sense), CATCATTAGCGTCGGTGAG (gal4 antisense), CATCGCGTCTCAGCCTCAC (NICD sense), CGGAATCGTTTATTGGTGTCG primer (NICD antisense), yielding a 1.2 or 0.3 kb amplification product, respectively.

All zebrafish experiments were approved by the national animal care committees (LANUV Nordrhein-Westfalen; 8.87-50.10.31.08.129; 84-02.04.2012.A251; City of Cologne; 576.1.36.6.3.01.10 Be) and the University of Cologne

### Lineage tracing

*Tg(krt19:creERT2)^fr35^*, *Tg(actb2:loxP-STOP-loxP-dsREDEx)^sd5^* double transgenic, *Tg(krt19:creERT2)^fr35^*, *Tg(actb2:loxP-STOP-loxP-dsREDEx)^sd5^*, *Tg(krt4:GFP)^gz7^* triple transgenic, or *Tg(krt4:creERT2)^fr33^*, *Tg(actb2:loxP-STOP-loxP-dsREDEx)^sd5^*; *Tg(krt4:GFP)^gz7^* triple transgenic embryos were treated with 5 µM 4-Hydroxytamoxifen (Sigma Aldrich; H7904) in the dark at 28°C from 24 hpf to 96 hpf before being returned to normal system conditions for growing up. For Figure 1A–D, larvae or adult fish were fixed with 4% paraformaledehyde (PFA)/PBS overnight at 4°C, followed by cryosectioning, mounting of sections in Mowiol (Carl Roth) containing DAPI and fluorophore analysis with a Zeiss Apotome. For Figure 1E fish were stained with rabbit anti-RFP (1∶100; MBL, PM005) (secondary = Alexa Fluor-488 anti-rabbit (1∶100; Invitrogen, A11008)), mouse anti-p63 (1∶100, Santa Cruz, sc-8431) (secondary = Alexa Fluor-647 anti-mouse (1∶100; Invitrogen, A21240)) and Rhodamine-Phalloidin (1∶100, Invitrogen, R415), and analyzed via confocal microcopy (Zeiss LSM710 Meta).

### Tissue-labelling procedures

Epidermal cell proliferation was assessed by BrdU incorporation after incubating adult fish in 100 µg/ml BrdU (Sigma) in fish system water for 12 or 24 hours, followed by anti-BrdU immunolabelling. TUNEL assay was performed using the *in situ* Cell Death Detection Kit, POD (Roche) according to the manufacturers recommendations.

For *in vivo* calcein staining, fish were incubated for two hours in calcein green or calcein blue solution (100 mg/l; Sigma Aldrich). After extensive washings, fish were anaesthetized with Tricaine (ethyl-3-aminobenzoate methanesulfonate, Fluka) for fluorescence analysis of live whole mounts or after PFA fixation and cryosectioning.

For histological, immunofluorescence and in situ hybridization analyses, adult zebrafish were sacrificed by Tricaine overdose and fixed in 4% PFA overnight at 4°C. Samples for paraffin embedding were decalcified in 0.5 M EDTA (pH 7.4) at room temperature for 5 days, dehydrated in a graded series of alcohols, cleared in Roti-Histol (Carl Roth) and embedded in paraffin wax. 10 µm sections were cut using a Leica RM2255 microtome. Samples for cryosections were orientated in 15% sucrose with 1% agarose in PBS and mounted in tissue freezing medium (Leica). 10 or 12 µm sections were obtained using a Leica CM1850 cryostat.

Paraffin sections were stained with hematoxylin & eosin or acidic fuchsin orange G (AFOG) trichrome (Gennova) according to standard protocols. For immunofluorescence analysis of paraffin- or cryosections, antigen retrieval was performed with 10 mM sodium citrate (pH 6.0) at 70°C for two hours, followed by washes and primary and secondary antibody incubations in PBS supplemented with 10% fetal calf serum (FCS), and mounting of sections in Mowiol containing DAPI. Primary antibodies other from the ones described above were: rabbit anti-activated caspase3 (1∶1000, abcam ab-13847), mouse anti-BrdU (1∶200, Roche 1170376), mouse anti-pan Keratin Type II (1∶200, Progen 61006). Secondary antibodies used were: anti-mouse Cy3 (1∶1000, Invitrogen), anti-rabbit Cy3 (1∶1000, Invitrogen), Alexa Fluor-488 anti-rabbit (1∶1000, Invitrogen)

In situ hybridization on paraffin sections was performed according to [80]. Antisense RNA probes were generated via in vitro transcription with Dig RNA labelling mix (Roche) and the following templates and conditions: *krt8* (GenBank BI875660): 1.8 kb cDNA fragment cloned from EST into pBluescript SK, linearization with *Hin*dIII, transcription with T3 RNA polymerase; *cki* (GenBank AF197880): 0.6 kb fragment in pSPORT, *Eco*RI, SP6 RNA pol; *krt5* (GenBank AF197909): 0.4 kb fragment in pBluescript SK, *Kpn*I, T3 RNA pol; *krt17* (ZFIN-ID zgc:92061; GenBank BI850052): 1.5 kb fragment in pSPORT, *Eco*RI, SP6 RNA pol. For *krtt1c11a* and *tgm1*, 1.0 kb fragments were amplified via RT-PCR and cloned into pGMTeasy (Promega) (*krtt1c11a: Spe*I, T7 RNA pol) or pCRII (*tgm1*: *Xho*I, SP6 RNA pol). For a TA-specific TAp63 probe, a 408 bp TA1 cDNA fragment was amplified via RT-PCR with the primers 5′-CAGGGGCTAGCTTCTAGTGG-3′ (sense) and 5′-TGTAAGGGGCTCCTCAGGCTC-3′ (antisense) and cloned into pGEMTeasy. The plasmid was digested with *Spe*I and transcribed with SP6 RNA pol for antisense, and with *Nco*I and T7 RNA pol for sense probe. For p53, EST clone MPMGp609B127Q8 with the full-length p53 cDNA in pSPORTI was linearized with *Eco*RI and transcribed with SP6 RNA pol for antisense, and with *Bam*HI and T7 RNA pol for sense probe.

Images were captured on a Zeiss Axiophot, Zeiss Apotome, Zeiss Confocal (LSM710 META) or Leica M165 FC stereo microscope.

### Electron microscopy

Transmission electron microscopy (TEM) of adult zebrafish was carried out as described [81]. For Scanning electron microscopy (SEM), adult fish were sacrificed and fixed overnight in 4% PFA at 4°C, dehydrated and either cryo-fixed, sputter-coated (gold/palladium) and transferred onto the SEM cryo-stage while still frozen, or critical point dried (CPD), sputter-coated and evaluated at room temperature.

### Chemical treatments

Fish were raised from 20–50 dpf in E3-medium containing 100 µg/ml of the γ-secretase inhibitor DAPT (N-N-(3,5-difluorophenacetyl)-L-alanyl)-S-phenylglycien t-butylester; Sigma-Aldrich 208255) [82], 5 µg/ml of the caspase 3 peptide inhibitor z-DEVD-fmk (Calbiochem 264155-80) [83] or 0.2% DMSO as control. Standard length (SL) of the fish was used to control equal development of each group.

### RT-PCR and RFLP analysis

RNA of whole zebrafish embryos at different developmental time points or from isolated tissues or organs of adults was isolated using the trizol reagent (Invitrogen). cDNA was generated using random hexamer primers. Regular PCR was carried out with the TA-specific sense primers TAS1-3 or the ΔN-specific sense primer ΔNs (see Figure S1), combined with a shared reverse primer 5-GTGACTGGGTGGGGCTATTT-3. Zebrafish *actb2* (GenBank: BC0675676) specific primers were used as control (sense, 5′-AGTTTGAGTCGGCGTGAAGT-3′; antisense, 5′-AGGCTGTGCTGTCCCTGTAT-3′). PCR reactions were performed with an annealing temperature of 55°C for 35 cycles.

For cDNA RFLP analysis, the 629 bp fragment shown in Figure 6B was amplified with primers TA3 and the reverse primer 5′-GTGACTGGGTGGGGCTATTT-3′, followed by overnight digest with *Mbo*I (NEB) and electrophoresis in 4% agarose gel, revealing 379, 107, 101 and 42 bp cleavage products in wild-type, but only 279, 208 and 42 bp products in hu2525 mutant cDNA.

Quantitative RT-PCR was performed in triplicates (2 experiments each) with TaqMan primers (see below) and an Applied Biosystems 7500 Fast Real-Time PCR System under default PCR conditions, resulting in specific 65 bp (TAp63; shared by TA1 and TA4) and 72 bp (ΔNp63) products. Used primers were: TA-forward, 5′-GCCTGAGGAGCCCCTTACA-3′; ΔN-forward, 5′-CCAATGCTCCCTCATCCTACA-3′, TA-reverse and ΔN-reverse, 5′-CATTTTGATCCATGCTGTTGAGA-3′; TA-TaqMan probe, 5′-CTCAGTATACAAGCCTGGG-3′; ΔN-TaqMan probe, 5′-AGCCTCAGTATACAAGCC-3′; standard, rps23 (ribosomal protein S23; standard; Applied Biosystems; Dr.0343030371m1). Amplification efficiencies were determined with a dilution series of cDNA from adult skin, and were above 95% for all three amplificants (TAp63, 99,4%; ΔNp63, 95.9%; rps23, 95.2%). Data were analyzed using Biosystems Prism SDS and Excel software, applying ΔCT and ΔΔCT calculations.

### Generation of expression constructs, mRNA synthesis and microinjection

To generate the TAp63 expression constructs pCS2-TA(1)p63γ and pCS2-TA(4)p63γ, a replacement strategy was used, amplifying the N-terminal fragments of the TA1 and TA4 isoforms of TAp63 via RT-PCR from adult skin of wild-type and hu2525 mutants with forward primers 5′-TTGGATCCACCATGACCTCTCCTTATGCAGC-3′ (TA1) or 5′-TTGGATCCACCATGTCACAGGGCCAGGGCTC-3′ (TA4), and reverse primer 5′-GTGACTGGGTGGGGCTATTT-3′, followed by *Bam*H1/*Bsp*M1 digest and cloning into *Bam*H1/*Bsp*M1-digested pCS2-ΔNp63γ [58]. To generate expression constructs for TAp63 with six N-terminal Myc tags, TAp63γ coding sequences were amplified from wild-type and hu2525 mutant pCS2-TA(4)p63γ plasmids with primers 5′-CGAATTCAACCATGTCACAGGGCCAGGGCTC-3′ (sense) and 5′- TTTCTAGATCACACTGATTGAGAACTCTTTTT G-3′ (antisense), digested with *Eco*RI and *Xba*I, and cloned into *Eco*RI/*Xba*I digested pCS2-MT (www.addgene.org/vector-database/2296/). For expression constructs with six C-terminal Myc tags, amplification was performed with primers 5′-TTGGATCCACCATGTCACAGGGCCAGGGCTC-3′ (sense) and 5′-CGATCGATTCACTGATTGAGAACTCTTTTTGTC-3′, followed by digestion with *Bam*HI and *Cla*I, and cloning into *Bam*HI/*Cla*I, digested pCS2-MT.

Capped RNA was prepared after restriction digest of these expression constructs or pCS2-ΔNp63α1 [58] with *Kpn*I, using the Message Machine kit (Ambion, Austin, TX). RNA was dissolved in water, and 1 nl per embryo injected. TAp63γ mRNAs were injected at a concentration of 10 ng/µl, 6xMyc-TAp63γ mRNAs at a concentration of 5 ng/µl, and ΔNp63α1 mRNA at a concentration of 25 ng/µl. Apoptosis and resulting embryonic death or embryonic malformations were scored at 8 hpf and 24 hpf, respectively, ΔNp63α1-induced loss of eyes at 32 hpf, as described [58].

### Immunoblotting

Zebrafish embryos were dechorionated and deyolked, and cells were collected as described [84]. Cell pellets or adult tissues were either directly dissolved in SDS loading buffer as described [84], or first lysed in chilled CSH buffer (50 mM Tris-HCl (pH 7.5), 250 mM NaCl, 1 mM EDTA, 1% Triton-X100, supplemented with cOmplete Protease Inhibitor Cocktail, Roche), followed by protein concentration determination. 10–12% SDS-PAGE, blotting on nitrocellulose membrane, Ponceau staining and immunodetection were carried out as described [84]. Used primary antibodies were: anti-Myc, 9B11 (mouse, Cell Signaling Technology; 1∶2000); anti-p63, 4A4 (mouse, Santa Cruz Technologies, against aa 1–205 of human ΔNp63), D-9 (mouse, Santa Cruz Biotechnology, against aa 15–151 of human ΔNp63), H-137 (rabbit, Santa Cruz Biotechnology, against aa 15–151 of human ΔNp63), H-129 (rabbit, Santa Cruz Biotechnology, against aa 513–641 at C-terminus of human TAp63α).

## Supporting Information

Figure S1 cDNA sequences of zebrafish TAp63 isoforms. Nucleotide and predicted amino acid sequences of the N-terminal region of the TA1 and TA4 isoforms of zebrafish TAp63 and of zebrafish ΔNp63 (GenBank accession number NM_152986). TAp63 sequences were derived from ENSDARG00000044356 (Ensembl), with the addition of TA1-specific exon 2, which was formerly not annotated. Sequences were confirmed via RT-PCR and DNA sequencing of amplified fragments. GenBank accession numbers are: TA(1)TAp63, KF682365; TA(1)TAp63, KF682366. Used sense primers for RT-PCR analyses are indicated in yellow. Exons are indicated in alternating colours (blue, black and red for alternative, ΔNp63-specific first exon). Start codons of TA1 and TA4 are in red, in-frame upstream stop codon of TA1 in orange; start codon and in-frame upstream stop codon of ΔNp63 are indicated in green. The nonsense mutation of the used TA-selective hu2525 allele is indicated in bold black letters.
(TIF)

Figure S2 Phylogenetic conservation of TAp63. (A,B) Multiple alignments of the TA-specific parts of the longer TA1 isoform of TAp63 from human, mouse and zebrafish (A) and of the shorter TA4 isoform of TAp63 from indicated vertebrate species ranging from human to shark, using the Jotun Hein method (DNAstar, Megalign). Amino acid residues identical to the human sequence are in red, similar residues in green. Please note that in mammals, the initially described TA4 isoform [18] has stronger transactivation activity than the later identified longer isoforms, including TA1, comparable to that of p53 [25]. (C) Phylogenetic tree of TA-specific part of the TA4 isoform of TAp63, generated by ClustalW method (DNAstar, Megalign). NCBI or Ensembl GenBank accession numbers of used sequences are: NM_003722.4 (human), NM_001127259.1 (mouse), ENSGALT00000011850 (chick), XM_002934050.1 (Xenopus tropicalis), ENSTNIT00000016990 (tetraodon), ENSTRUT00000008073 (fugu), ENSORLT00000019617 (Medaka), JN794074.1 (elephant shark).
(TIF)

Figure S3 TAp63 sequencing profiles of hu2525 mutants and wild-type siblings. (A) wild-type sibling; (B) heterozygous animal; (C) homozygous mutant. In (A) and (C), the encoded amino sequence is indicated.
(TIF)

Figure S4 Overview images of H&E staining and p63 immunofluorescence in TAp63 and p53 mutant and wild-type sibling zebrafish. (A–D) Hematoxylin & eosin staining; transverse sections through breeding tubercle disc region of lower jaw of wild-type (A), TAp63 mutant (B; strong phenotype; C2), TAp63 mutant (C; intermediate phenotype; C1) and p53 mutant (I; intermediate phenotype; C1) at 1 year. For higher magnifications of breeding tubercle regions of panels (A,B,D), see Figure 7G–I. (E–G) anti-p63 immunofluorescence; transverse sections through lower jaw of wild-type (E), TAp63 mutant (F) and p53 mutant (G); 1 year of age. Breeding tubercle disc region is indicated by arrow, breeding tubercle row region by arrowhead. Note that both mutants lack breeding tubercles in the row region, which consists of multiple p63-positive layers. For higher magnifications of disc-shaped breeding tubercle regions of panels (E,F), see Figure 7A,B.
(TIF)

Figure S5 Differential effects of DAPT and zDEVD on Notch signalling are statistically significant.(A) Graphic illustration of distribution of standard body lengths of tg(TP1bglob:egfp) Notch reporter fish at 50 dpf, after treatment with DMSO, DAPT or zDEVD from 20–50 dpf. Inhibitor treatments had no effects on somatic growth of fish. Numbers of evaluated fish and standard deviations are indicated. (B) Graphic illustration of percentages of tg(TP1bglob:egfp) Notch reporter fish at 50 dpf with GFP (left columns) or calcein blue signals (right columns) in lower jaw breeding tubercles, after treatment with DMSO, DAPT or zDEVD from 20–50 dpf and incubation in blue calcein. Both DAPT- and zDEVD treatment leads to loss of calcein-positive cells (column 5, 87.5%; column 6, 60%), whereas expression of the Notch reporter is only lost upon DAPT treatment (column 2, 68.75%), but not upon zDEVD treatment (column 3, 0%). Numbers of evaluated fish are indicated.
(TIF)
