# Supplementary figures and images for: p53 and TAp63 Promote Keratinocyte Proliferation and Differentiation in Breeding Tubercles of the Zebrafish
Source: PLoS Genet. 2014 Jan 9;10(1):e1004048. doi: 10.1371/journal.pgen.1004048 (PMC3886889; doi:10.1371/journal.pgen.1004048)

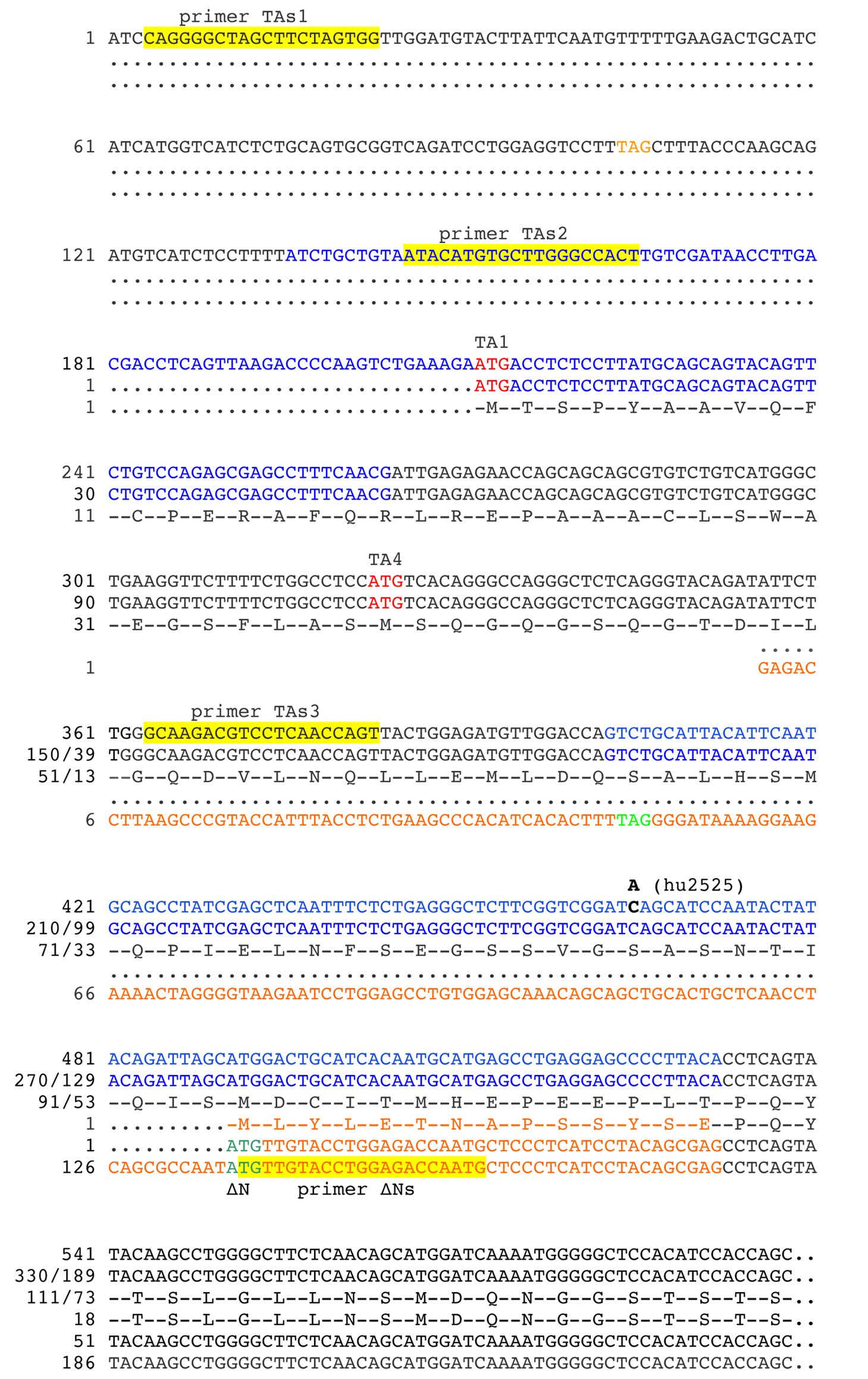

Supplement: Figure S1 — cDNA sequences of zebrafish TAp63 isoforms. Nucleotide and predicted amino acid sequences of the N-terminal region of the TA1 and TA4 isoforms of zebrafish TAp63 and of zebrafish ΔNp63 (GenBank accession number NM_152986). TAp63 sequences were derived from ENSDARG00000044356 (Ensembl), with the addition of TA1-specific exon 2, which was formerly not annotated. Sequences were confirmed via RT-PCR and DNA sequencing of amplified fragments. GenBank accession numbers are: TA(1)TAp63, KF682365; TA(1)TAp63, KF682366. Used sense primers for RT-PCR analyses are indicated in yellow. Exons are indicated in alternating colours (blue, black and red for alternative, ΔNp63-specific first exon). Start codons of TA1 and TA4 are in red, in-frame upstream stop codon of TA1 in orange; start codon and in-frame upstream stop codon of ΔNp63 are indicated in green. The nonsense mutation of the used TA-selective hu2525 allele is indicated in bold black letters. (TIF) [file pgen.1004048.s001.tif]

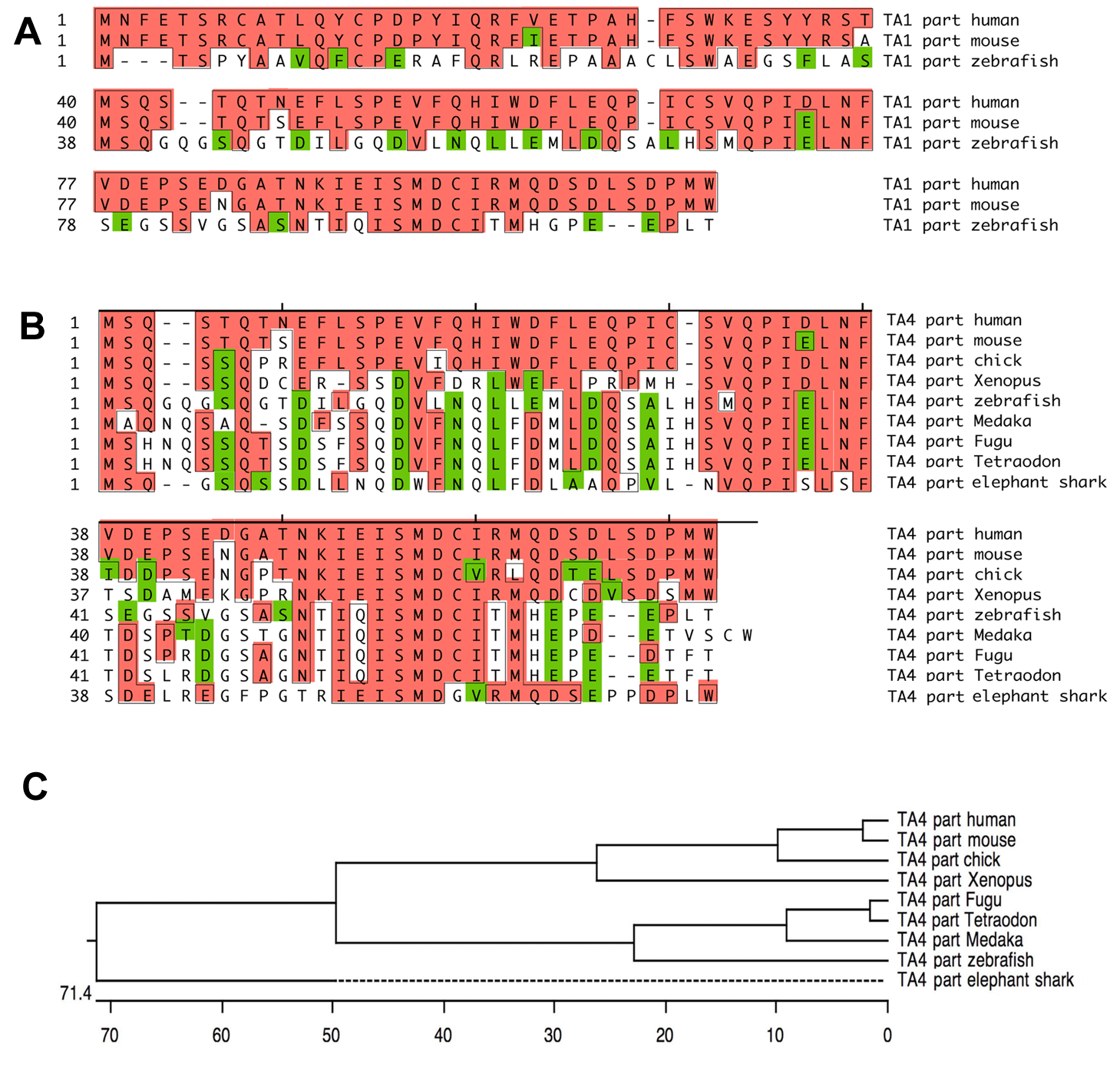

Supplement: Figure S2 — Phylogenetic conservation of TAp63. (A,B) Multiple alignments of the TA-specific parts of the longer TA1 isoform of TAp63 from human, mouse and zebrafish (A) and of the shorter TA4 isoform of TAp63 from indicated vertebrate species ranging from human to shark, using the Jotun Hein method (DNAstar, Megalign). Amino acid residues identical to the human sequence are in red, similar residues in green. Please note that in mammals, the initially described TA4 isoform [18] has stronger transactivation activity than the later identified longer isoforms, including TA1, comparable to that of p53 [25]. (C) Phylogenetic tree of TA-specific part of the TA4 isoform of TAp63, generated by ClustalW method (DNAstar, Megalign). NCBI or Ensembl GenBank accession numbers of used sequences are: NM_003722.4 (human), NM_001127259.1 (mouse), ENSGALT00000011850 (chick), XM_002934050.1 (Xenopus tropicalis), ENSTNIT00000016990 (tetraodon), ENSTRUT00000008073 (fugu), ENSORLT00000019617 (Medaka), JN794074.1 (elephant shark). (TIF) [file pgen.1004048.s002.tif]

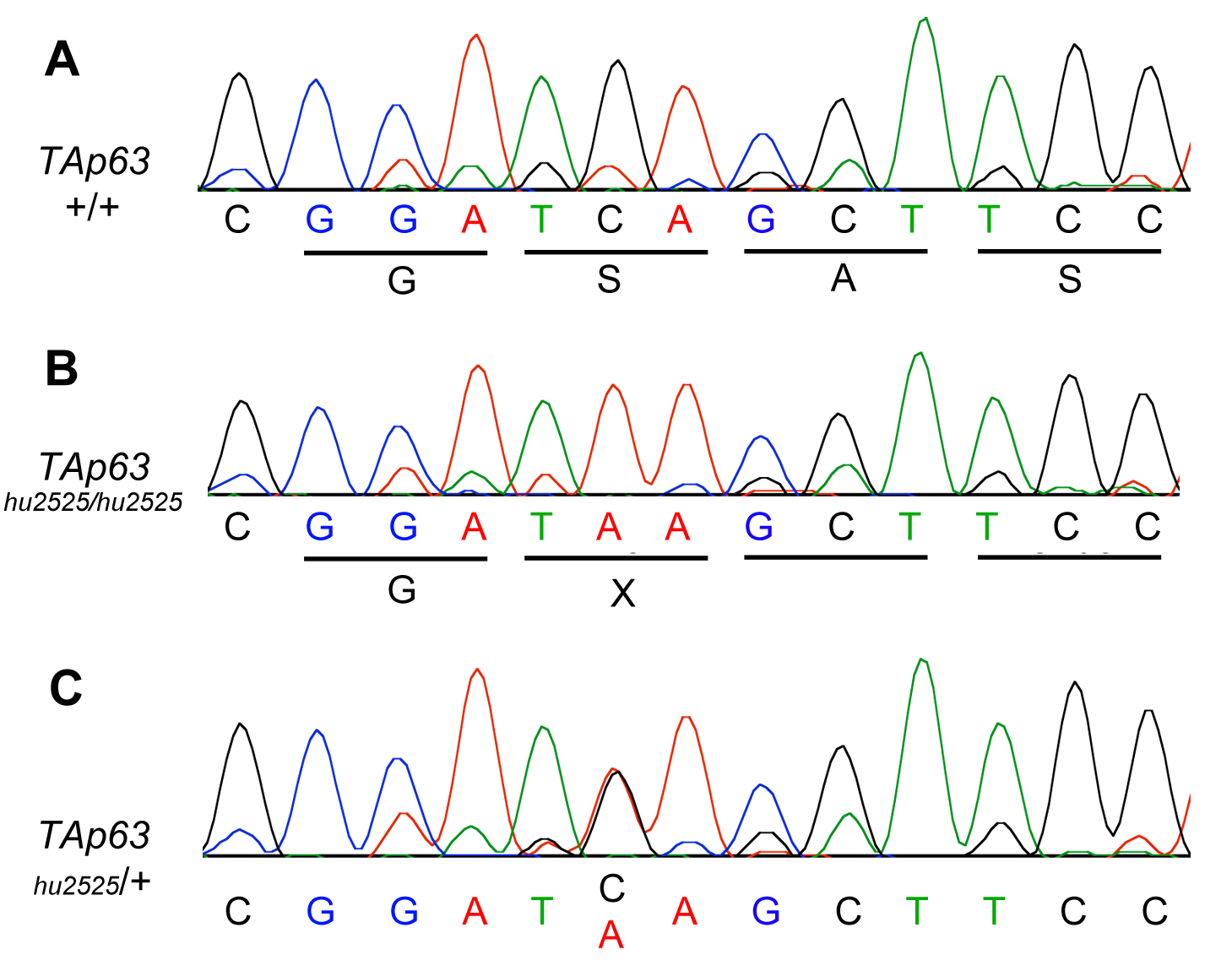

Supplement: Figure S3 — TAp63 sequencing profiles of hu2525 mutants and wild-type siblings. (A) wild-type sibling; (B) heterozygous animal; (C) homozygous mutant. In (A) and (C), the encoded amino sequence is indicated. (TIF) [file pgen.1004048.s003.tif]

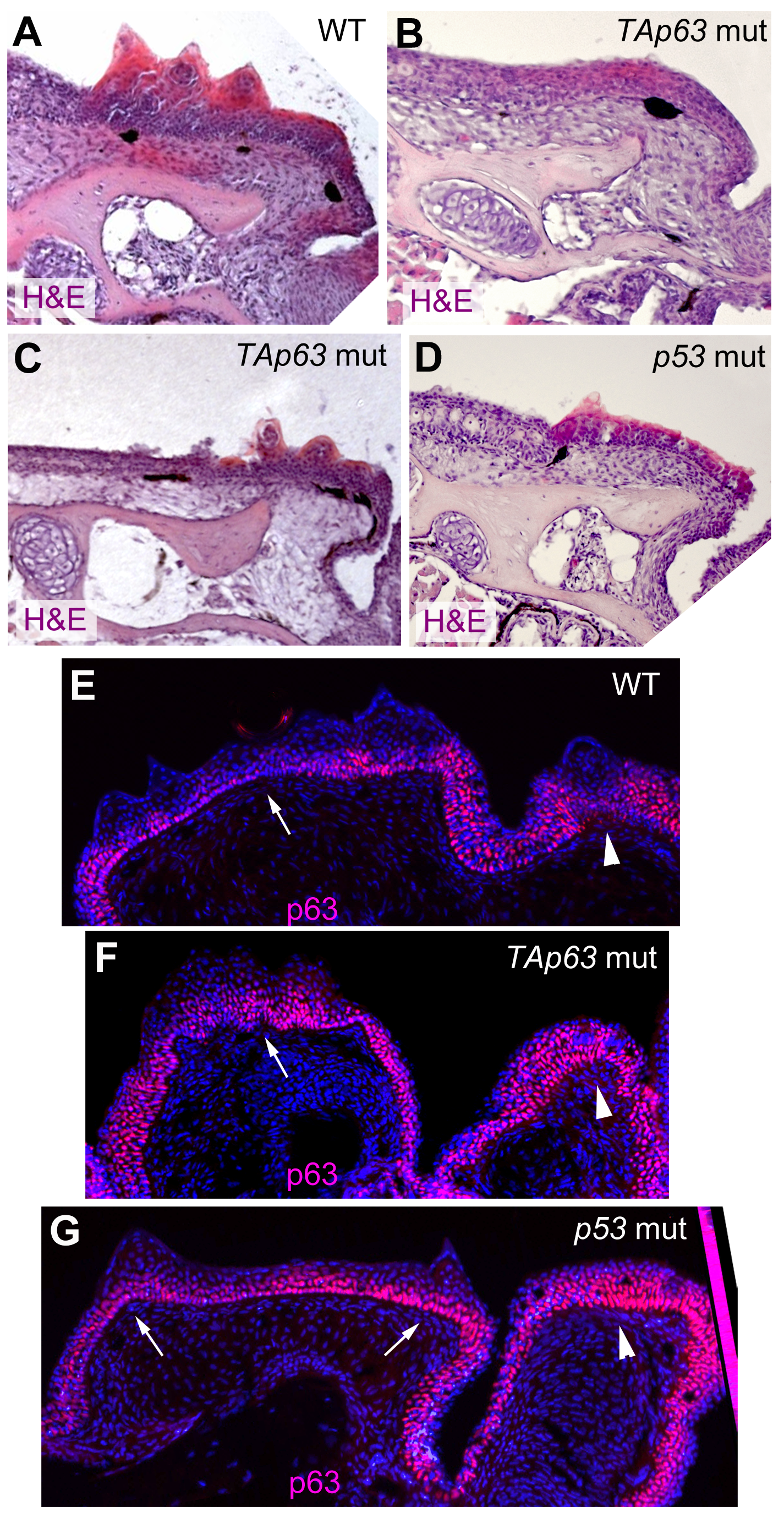

Supplement: Figure S4 — Overview images of H&E staining and p63 immunofluorescence in TAp63 and p53 mutant and wild-type sibling zebrafish. (A–D) Hematoxylin & eosin staining; transverse sections through breeding tubercle disc region of lower jaw of wild-type (A), TAp63 mutant (B; strong phenotype; C2), TAp63 mutant (C; intermediate phenotype; C1) and p53 mutant (I; intermediate phenotype; C1) at 1 year. For higher magnifications of breeding tubercle regions of panels (A,B,D), see Figure 7G–I. (E–G) anti-p63 immunofluorescence; transverse sections through lower jaw of wild-type (E), TAp63 mutant (F) and p53 mutant (G); 1 year of age. Breeding tubercle disc region is indicated by arrow, breeding tubercle row region by arrowhead. Note that both mutants lack breeding tubercles in the row region, which consists of multiple p63-positive layers. For higher magnifications of disc-shaped breeding tubercle regions of panels (E,F), see Figure 7A,B. (TIF) [file pgen.1004048.s004.tif]

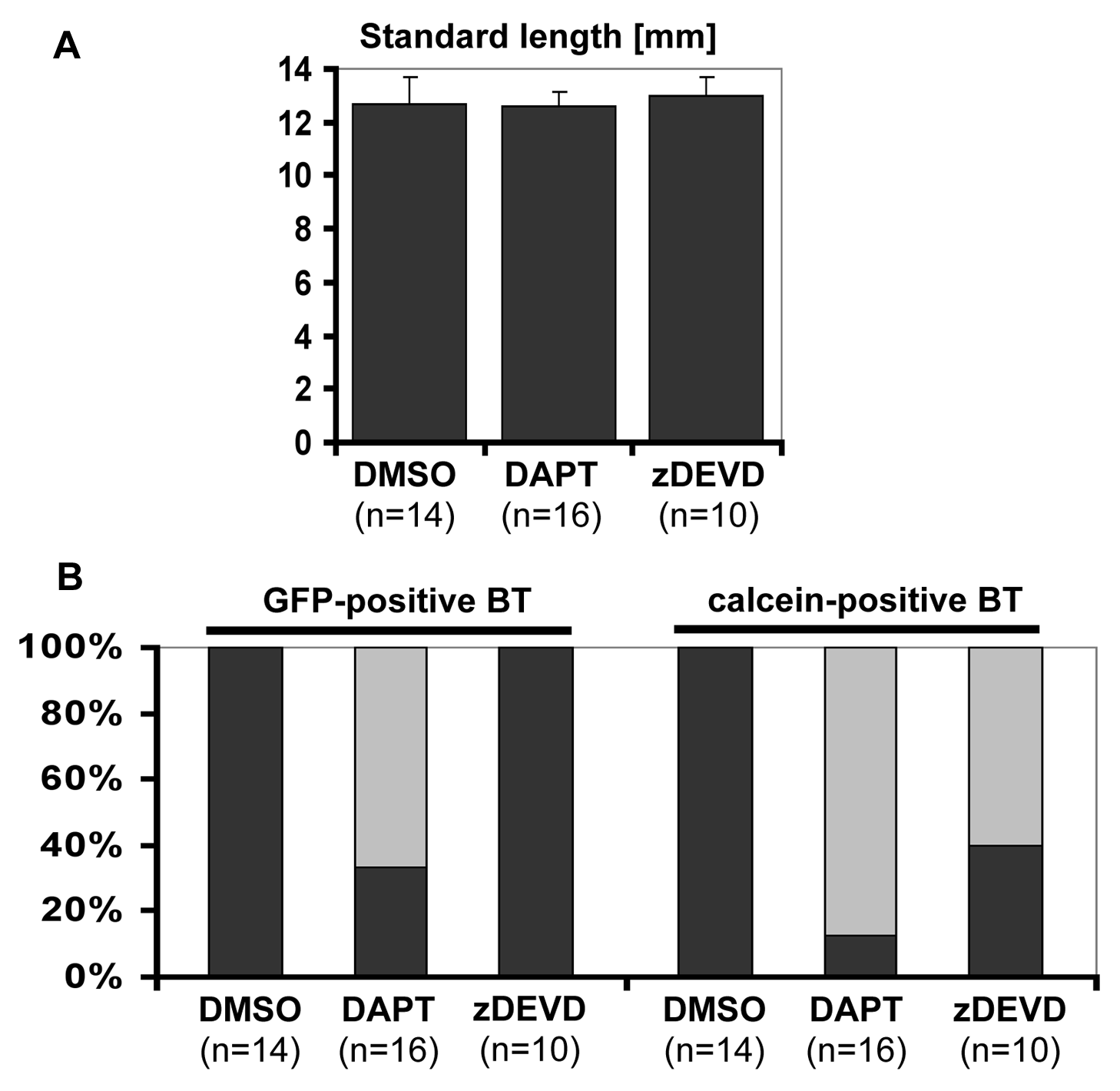

Supplement: Figure S5 — Differential effects of DAPT and zDEVD on Notch signalling are statistically significant.(A) Graphic illustration of distribution of standard body lengths of tg(TP1bglob:egfp) Notch reporter fish at 50 dpf, after treatment with DMSO, DAPT or zDEVD from 20–50 dpf. Inhibitor treatments had no effects on somatic growth of fish. Numbers of evaluated fish and standard deviations are indicated. (B) Graphic illustration of percentages of tg(TP1bglob:egfp) Notch reporter fish at 50 dpf with GFP (left columns) or calcein blue signals (right columns) in lower jaw breeding tubercles, after treatment with DMSO, DAPT or zDEVD from 20–50 dpf and incubation in blue calcein. Both DAPT- and zDEVD treatment leads to loss of calcein-positive cells (column 5, 87.5%; column 6, 60%), whereas expression of the Notch reporter is only lost upon DAPT treatment (column 2, 68.75%), but not upon zDEVD treatment (column 3, 0%). Numbers of evaluated fish are indicated. (TIF) [file pgen.1004048.s005.tif]
